# Docosahexaenoic acid (DHA) effects on proliferation and steroidogenesis of bovine granulosa cells

**DOI:** 10.1186/s12958-018-0357-7

**Published:** 2018-04-26

**Authors:** Virginie Maillard, Alice Desmarchais, Maeva Durcin, Svetlana Uzbekova, Sebastien Elis

**Affiliations:** 1 UMR PRC, CNRS, IFCE, INRA, Université de Tours, 37380 Nouzilly, France; 2 INRA Centre Val de Loire, Physiologie de la Reproduction et des Comportements, 37380 Nouzilly, France

**Keywords:** Bovine, Gene expression, Lipid, N-3 PUFA, DHA, Folliculogenesis, MAPK, AKT, AMPK, Signaling pathways, Free fatty acid receptor 4 (FFAR4)

## Abstract

**Background:**

Docosahexaenoic acid (DHA) is a n-3 polyunsaturated fatty acid (PUFA) belonging to a family of biologically active fatty acids (FA), which are known to have numerous health benefits. N-3 PUFAs affect reproduction in cattle, and notably directly affect follicular cells. In terms of reproduction in cattle, n-3 PUFA-enriched diets lead to increased follicle size or numbers.

**Methods:**

The objective of the present study was to analyze the effects of DHA (1, 10, 20 and 50 μM) on proliferation and steroidogenesis (parametric and/or non parametric (permutational) ANOVA) of bovine granulosa cells in vitro and mechanisms of action through protein expression (Kruskal-Wallis) and signaling pathways (non parametric ANOVA) and to investigate whether DHA could exert part of its action through the free fatty acid receptor 4 (FFAR4).

**Results:**

DHA (10 and 50 μM) increased granulosa cell proliferation and DHA 10 μM led to a corresponding increase in proliferating cell nuclear antigen (PCNA) expression level. DHA also increased progesterone secretion at 1, 20 and 50 μM, and estradiol secretion at 1, 10 and 20 μM. Consistent increases in protein levels were also reported for the steroidogenic enzymes, cytochrome P450 family 11 subfamily A member 1 (CYP11A1) and hydroxy-delta-5-steroid dehydrogenase, 3 beta- and steroid delta-isomerase 1 (HSD3B1), and of the cholesterol transporter steroidogenic acute regulatory protein (StAR), which are necessary for production of progesterone or androstenedione. FFAR4 was expressed in all cellular types of bovine ovarian follicles, and in granulosa cells it was localized close to the cellular membrane. TUG-891 treatment (1 and 50 μM), a FFAR4 agonist, increased granulosa cell proliferation and MAPK14 phosphorylation in a similar way to that observed with DHA treatment. However, TUG-891 treatment (1, 10 and 50 μM) showed no effect on progesterone or estradiol secretion.

**Conclusions:**

These data show that DHA stimulated proliferation and steroidogenesis of bovine granulosa cells and led to MAPK14 phosphorylation. FFAR4 involvement in DHA effects requires further investigation, even if our data might suggest FFAR4 role in DHA effects on granulosa cell proliferation. Other mechanisms of DHA action should be investigated as the steroidogenic effects seemed to be independent of FFAR4 activation.

**Electronic supplementary material:**

The online version of this article (10.1186/s12958-018-0357-7) contains supplementary material, which is available to authorized users.

## Background

N-3 polyunsaturated fatty acids (PUFAs) belong to a family of biologically active fatty acids (FAs) and are known to have numerous health benefits [1]. The most well-known members are alpha-linolenic acid (ALA), eicosapentaenoic acid (EPA) and docosahexaenoic acid (DHA). These are essential FAs, as mammals cannot produce ALA, the precursor of n-3 PUFA. Furthermore, DHA, the longest-chain member of this family, has a 22-carbon chain (C22:6) and can theoretically be produced from shorter-chain members of this family. As the efficiency of the desaturation reaction is low, the most extensive source of DHA is the diet, cold-water fish in particular [2, 3]. DHA is reported to exert pleiotropic effects at both central and peripheral levels, including the brain, heart and immune system [4–6].

Among their physiological roles, n-3 PUFAs affect reproduction in cattle [7, 8]. The feeding of diets supplemented with n-3 PUFAs (enriched in ALA) was shown to increase calving rate in dairy cows [9–11]. At the uterus level, n-3 PUFAs reduced prostaglandin F2 alpha (PGF2α) secretion from endometrial cells [12–15]. Moreover, n-3 PUFA dietary supplement in cows was reported to exert effects at the ovarian level. Indeed, a n-3 PUFA-enriched diet led to an increased cleavage rate or a trend to increased blastocyst rate after in vivo maturation [16, 17] and n-3 PUFA supplementation during in vitro maturation also led to increased blastocyst rates [18, 19]. In addition, dietary ALA supplementation led to an increase in the size of the pre-ovulatory follicles [9], in number of total follicles [16] or small follicles [17], to larger corpus luteum size and higher plasma progesterone levels [20, 21]. Dietary fish oil supplementation, containing both EPA and DHA, also led to an increased number of large follicles [11]. Dietary DHA improved resumption of estrous cyclicity and pregnancy at first artificial insemination [22]. These data suggest that n-3 PUFAs could favorably affect folliculogenesis by having direct effects on follicular cells, in addition to the already described effects on the oocyte. Nevertheless, several studies reported no effect of n-3 PUFA diet on female reproduction [21, 23], or even a deleterious effect leading to a more unfavorable uterine environment for sustaining pregnancies and reduced fertility in cows [24].

There are several mechanisms by which n-3 PUFAs could exhibit multiple physiological roles in organism development and diseases [25]: indirect actions on cells by influencing metabolite, hormone concentration, or other factors as oxidation of LDL and oxidative stress, direct actions on cells via receptors, sensors or cell membrane fatty acid composition (membrane order, lipid rafts, etc.) (reviewed in [26]). Most of the studies of n-3 PUFA mechanisms concern the inflammatory process [27]. Indeed, n-3 PUFAs were shown to affect cytokine expression, especially inflammatory cytokines (TNF, IL-1β, IL-6 and IL-8), and to simultaneously help produce inflammatory resolving metabolites (resolvins, protectins, maresins) [26, 28]. Feeding with a n-3 PUFA-enriched diet results in the membrane lipid composition being enriched in n-3 PUFA. Eicosanoids (prostaglandins, leucotrienes, thromboxanes and lipoxins) produced from n-3 PUFAs (for example, 3-series prostaglandins) are therefore increased and, conversely, eicosanoids produced from arachidonic acid (for example, 2-series prostaglandins) are reduced [28]. This decrease in 2-series prostaglandins induced a less inflammatory environment because 3-series prostaglandins are less inflammatory [29]. N-3 PUFAs were also reported to act through intracellular sensors such as peroxisome proliferator-activated receptors (PPARs) and nuclear factor kappa B and thus to modulate their target gene expression [26]. By affecting the membrane lipid composition, n-3 PUFAs are also able to increase membrane fluidity and to promote lipid raft formation and organization (reviewed in [30]), and therefore signaling pathways. N-3 PUFAs are also reported to act through free fatty acid receptors, FFAR1 and FFAR4, located in the membrane and activate their signaling pathways [31]. Indeed, FFAR4 can modulate signaling of mitogen-activated protein kinases MAPK1/3 (alias ERK 1/2) and MAPK14 (alias p38), and protein kinase B (Akt) [32, 33], which are already known to be involved in bovine granulosa cell (GC) proliferation and steroidogenesis [34–38].

In the present study, we choose to focus solely on DHA. Indeed, recent in vivo studies focused on algae oil supplementation, containing mainly DHA and no EPA (substitute of fish oil which does not exhibit the limitation of fish oil: limited resources and competition with human diet) [22, 39, 40]. Studying solely DHA effects is therefore a relevant step to understand its mechanism of action granulosa cells. We hypothesize that DHA could stimulate steroidogenesis in bovine GC and that it could exert part of its activity through the membrane receptor, FFAR4. The objective of the present study was therefore to examine the effects of DHA on proliferation and steroidogenesis of bovine GCs. We then investigated FFAR4 expression in GCs and reported its expression in ovarian follicles and in GCs. We thus investigated whether supplementation of the culture medium with a FFAR4 agonist, TUG-891 [41], could reproduce the effects of DHA in GCs. Treatment with DHA and TUG-891 was performed during GC culture, followed by examination of cell proliferation, progesterone and estradiol secretion, gene and protein expression, and signaling pathways.

## Methods

### Ethics

No experiments with living animals were performed.

### Chemicals and antibody

Customized anti-FFAR4 antibody was produced from a rabbit immunized against a peptide designed from the second extracellular loop (179–194 amino acids) of the bovine sequence (Accession number: NP_001315586.1). Serum from immunized rabbit underwent affinity purification and antibody concentration was measured by enzyme-linked immunosorbent assay (ELISA; Agro-Bio, La Ferté Saint-Aubin, France). This customized antibody enabled detection of a 42 kDa band by western blot in lung tissue extract (Additional file 1: Figure S1). Lung tissue was used to validate the antibody, as in other species, namely mice and humans, the level of expression of FFAR4 protein is reported to be higher in this tissue than in other tissues, such as adipose tissue, intestine and liver [42].

DHA was obtained from Sigma-Aldrich (Saint Quentin Fallavier, France) and TUG-891 from Tocris (Bio-Techne Europe, Lille, France). Unless otherwise stated in the text, all other chemicals were obtained from Sigma-Aldrich (Saint Quentin Fallavier, France). Primary antibodies used are indicated in Additional file 2: Table S1. Horseradish peroxidase (HRP)-conjugated anti-rabbit and anti-mouse were purchased from Perkin Elmer (Courtaboeuf, France) and biotinylated horse anti-mouse and anti-rabbit IgG were from Vector Laboratories (Clinisciences, Nanterre, France). Alexa Fluor 488-conjugated donkey anti-rabbit IgG was purchased from Jackson ImmunoResearch (Newmarket, United Kingdom). Dimethyl sulfoxide (DMSO) 1/2000 was used as the control treatment as it is used as a solvent for DHA and TUG-891.

### Biological material

Ovarian cortex were carefully dissected from 5 bovine ovaries collected from a local slaughterhouse. Theca cells were retrieved from healthy follicles of 5 bovine ovaries (local slaughterhouse): once the follicle is carefully dissected and opened, the inside of the follicle is gently scraped and washed in PBS to remove granulosa cells and cumulus-oocyte complex. Cumulus cells (without oocyte) were recovered as previously described [19] from 20 ovaries, briefly cumulus-oocyte complexes were sorted out after follicular punctures and oocytes were dissociated from cumulus cells by mechanical aspiration and removed. GCs were collected according the same protocol as when performing cell culture experiment (described in the following section). In the case of GC culture, antral follicles with a diameter of 2 to 6 mm were used. Ovaries exhibiting very large corpora lutea were excluded from the samples to avoid contamination of our granulosa cell samples from growing follicles with luteinized granulosa cells (originating from these corpora lutea) during ovary punctures. Lung tissue was collected from one cow (local slaughterhouse).

### Isolation and culture of granulosa cells

Follicles from bovine ovaries were punctured using an 18G needle linked to a vacuum pump and a 50-mL falcon tube, enabling the collection of follicular fluid which contained GCs. GCs were then washed in modified McCoy’s 5A serum-free medium containing: L-glutamine (3 mM), HEPES (20 mM; pH 7.6), bovine serum albumin (BSA; essentially fatty acid free; 0.1%), penicillin-streptomycin (120 × 10^3^ UI/L penicillin; 120 mg/L streptomycin), amphotericin B (50 μg/L), bovine insulin (1.74 nM), bovine apo-transferrin (5 mg/L), selenium (0.12 μM) and 4-androsten-11β-ol-3,17-dione (96 nM). After centrifugation and washes in medium, suspended cells were dropped off on a Percoll density medium (50% Percoll, 50% medium) and GCs were purified by centrifugation (300 g). Recovered GCs were incubated in serum-free modified McCoy’s 5A medium in the appropriate cell density and plates according to the assays as described below. From the beginning of the culture, cells were cultured in the presence or absence of test reagents for different durations depending on the biological function of interest. Cultures were performed in a water-saturated atmosphere containing 5% CO_2_ in air at 38 °C. The use of culture media without fetal bovine serum and a short culture duration limited the differentiation of GCs, which retained the round shape exhibited in vivo. Under the culture conditions used in this experiment, GCs still exhibited steroidogenic activity and were able to proliferate. The Additional file 3: Figure S2 presents the experiment design of the present work.

### Immunohistochemistry

Bovine ovaries were embedded in paraffin and serially sectioned at a thickness of 7 μm using a microtome. Adjacent sections were deparaffinized in toluene, rehydrated and incubated in antigen unmasking solution (1% in water; Vector Laboratories) for 2 min in a microwave (850 W), left to cool for 1 h at room temperature, and washed twice in phosphate buffered saline (PBS) for 5 min. Endogenous peroxidase activity was quenched by treating sections with 0.3% hydrogen peroxide in PBS containing 0.1% triton for 30 min at 4 °C. After two 5-min washes in PBS, sections were incubated in PBS containing BSA (2%) and goat serum (5%) for 1 h at room temperature and quickly washed in PBS with BSA (0.1%). Sections were incubated overnight at 4 °C in PBS with BSA (0.5%) with primary rabbit antibodies against bovine FFAR4 (19 μg/mL; customized antibody; Agro-Bio) or with the pre-immunized serum (similar IgG concentration; Agro-Bio) from the same rabbit (negative control). After four 5-min washes in PBS with Tween 20 (0.1%), sections were then incubated with secondary biotinylated horse anti-mouse and anti-rabbit IgG (1:700 dilution) in PBS with BSA (0.5%) for 1 h at room temperature. After four 5-min washes in PBS with Tween 20 (0.1%), sections were incubated in a ready-to-use avidine and biotinylated horseradish peroxidase solution (Vectastain® Elite® ABC kit, Vector Laboratories) for 30 min at room temperature, according to the manufacturer’s instructions. After three 5-min washes in PBS with Tween 20 (0.1%), immunostaining was developed by incubating sections in 50 mM Tris-HCl (pH 7.8) containing 0.4 mg/ml 3,3′-diaminobenzidine tetrahydrochloride dehydrate (DAB) and 0.007% hydrogen peroxide for 5 min at room temperature. Sections were then dehydrated and mounted using Depex. Immuno-specific staining (brown) was observed using an Axioplan Zeiss transmission microscope coupled with a 10× objective with a numerical aperture of 0.03 or a 40× objective with a numerical aperture of 0.75. Images were generated with a numerical camera piloted by the Software Spot (version 5.2 for Windows; Diagnostic Instruments, Inc., MicroMecanique, Evry, France).

### Immunofluorescence

After collection and washes, as described above, GCs were incubated on a 8-well chamber slide (Lab-Tek® Nunc, Thermo-Fisher Scientific, Courtaboeuf, France) for 48 h. Cells were then washed in Tris-buffer saline (TBS), fixed in PBS containing paraformaldehyde (4%) for 20 min and washed in TBS-0.1% BSA for 3 min at room temperature. After blocking in TBS containing BSA (1%) and horse serum (5%) for 1 h at room temperature, GCs were incubated overnight at 4 °C in TBS containing BSA (1%) and horse serum (5%) with primary rabbit antibodies against bovine FFAR4 (9.5 μg/mL; customized antibody) or the pre-immunized serum (similar IgG concentration) of the same rabbit (negative control). Other GCs were incubated in the same conditions with primary rabbit antibodies against human FFAR4 (20 μg/mL; Aviva Systems Biology) or rabbit IgG (similar concentration) as a negative control. After three 10-min washes in TBS containing BSA (0.1%), sections were then incubated with secondary Alexa Fluor 488-conjugated donkey anti-rabbit IgG (1:800 dilution) in TBS containing BSA (0.1%) for 1.5 h at room temperature. Then, GCs were washed in TBS containing BSA (0.1%) (three 10-min washes), and incubated in TBS with 1 μg/mL Hoechst 33,258 for 15 min at room temperature to stain nuclei. Sections were then mounted using Moviol® and fluorescence was observed under a Zeiss confocal microscope LSM700 (Carl Zeiss Microscopy GmbH, Munich, Germany) using an oil 40× objective with a numerical aperture of 1.3 or an oil 63× objective with a numerical aperture of 1.4 and the appropriate filters. Images were captured using Zen 2012 software (black edition version 8.0, Carl Zeiss Microscopy GmbH).

### GC proliferation

Cell proliferation was determined by measurement of ^3^H-thymidine incorporation into bovine GCs after 24 h of culture, as previously described [43] with some modifications. Briefly, GCs (2.5 × 10^5^ viable cells/250  μL media/well) were cultured in 48-well dishes in modified McCoy’s 5A media containing ^3^H-thymidine (0.25nCi/μL corresponding to 18.5 kBq/mL; Perkin Elmer, Courtaboeuf, France) in the presence or absence of DHA (1, 10, 20 or 50 μM) or TUG-891 (1, 10 or 50 μM). Cultures were maintained at 38  °C in air containing 5% CO_2_. After culture for 24  h, excess thymidine was removed by washing twice with PBS (200 μL/well). Cells were then fixed with cold 50% trichloroacetic acid (100 μL/well) for 15 min and lysed using 0.5 M NaOH (250 μL/well) for 10 min at room temperature. Radioactivity was counted using Ultima Gold MV scintillation fluid (Perkin Elmer) and a β-photomultiplier C2900 (Perkin Elmer). The results are expressed as mean ± SEM of 13 independent cultures (cells from one culture came from several follicles originating from several ovaries (around 10 follicles per ovary, and 15 ovaries per cultures) and each culture came from a specific batch of ovaries), with four replicates of each treatment. Data are expressed as disintegrations per minute (dpm).

### Progesterone assay

GCs were cultured in 96-well dishes (1 × 10^5^ viable cells/150 μL media/well) in modified McCoy’s 5A medium in the presence or absence of DHA (1, 10, 20 or 50 μM) or TUG-891 (1, 10 or 50 μM) for 48 h. Supernatants and cells were separately stored at − 20 °C until progesterone analysis and protein assays (BCA protein quantification kit;Interchim, Montluçon, France), respectively. The concentration of progesterone was determined in the culture media using an ELISA protocol, as described previously [44]. For progesterone concentrations ranging from 0.25 to 32  ng/mL, the intra-assay coefficient of variation (CV) averaged < 10%. Progesterone secreted in each well was normalized by the protein concentration of the same well. The results are expressed as the amount of progesterone (ng/mL) secreted per 48  h per protein amount (μg/mL) per well. Data, representing 12 independent cultures (as described in GC-proliferation section) with each treatment conducted in quadruplicate, are expressed as mean ± SEM and as ng of secreted progesterone per μg of protein.

### Estradiol assay

GCs were cultured in 96-well dishes (1 × 10^5^ viable cells/150 μL media/well) in modified McCoy’s 5A medium in the presence or absence of DHA (1, 10, 20 or 50 μM) or TUG-891 (1, 10 or 50 μM) for 48 h. Supernatants and cell layer were separately stored at − 20 °C until estradiol analysis and protein assays (BCA protein quantification kit), respectively. The concentration of estradiol in the culture media was determined using the DIAsource E2-EASIA Kit (DIAsource, Louvain-la-Neuve, Belgium), in accordance with the manufacturer’s recommendations. Briefly, 50 μL of spent medium was used for the assay and the competition between unlabeled estradiol (present in the culture media) and labeled estradiol (provided by the kit) lasted overnight at 4 °C. For estradiol concentrations ranging from 1.56 to 50  pg/mL, the inter-assay CVs averaged 15%. Estradiol was normalized as described for progesterone. Data, represented six independent cultures with each treatment conducted in quadruplicate, are expressed as mean ± SEM as pg of secreted estradiol per μg of protein.

### Fatty acid analysis

GCs were cultured in 48-well dishes (2.5 × 105 viable cells/250 μL media/well) in modified McCoy’s 5A medium in the presence or absence of DHA (1, 10 or 50 μM) for 15 h. Supernatants were removed. Cells with the same treatment (pool of 12 wells) were isolated in PBS 1X and stored at − 80 °C until lipid analysis. Lipids were extracted and analyzed as previously described [45]. Briefly, total lipids were extracted twice from granulosa cells with ethanol/chloroform (1:2, *v*/v). Before extraction, 1,2-diheptadecanoyl-sn-gycero-3-phosphocholine (GPC di-17:0) was added as internal standard. Lipids were transmethylated with toluene-methanol (2:3, v/v) and boron trifluoride in methanol (14%) at 100 °C for 90 min in screw-capped tubes. After addition of 1.5 mL K2CO3 in 10% water, the resulting fatty acid methyl esters (FAME) were extracted by 2 mL of isooctane. The FAMEs were analyzed by gas chromatography with a HP6890 instrument equipped with a fused silica capillary BPX70 SGE column (60 × 0.25 mm). The vector gas was hydrogen. Temperatures of the Ross injector and the flame ionization detector were set at 230 °C and 250 °C, respectively. FAMEs were identified by making a comparison of their relative retention times with those of commercial standards. Fatty acid composition is expressed as the mole percentage of total fatty acids. The results are expressed as means ± SEM (*n* = 4 per treatment).

### Protein extraction and western blot analysis

In order to validate the FFAR4 antibody designed against the bovine protein, proteins were extracted from lung tissue. Moreover, on GC, assays were conducted for proliferating cell nuclear antigen (PCNA), for steroidogenic enzymes, cytochrome P450 family 11 subfamily A member 1 (CYP11A1 alias cholesterol side-chain cleavage) and hydroxy-delta-5-steroid dehydrogenase, 3 beta- and steroid delta-isomerase 1 (HSD3B1 alias 3β-HSD), and for the cholesterol transporter steroidogenic acute regulatory protein (StAR). GCs were cultured in 48-well dishes (2.5 × 10^5^ viable cells/250 μL media/well) in modified McCoy’s 5A medium in the presence or absence of DHA for 15 h. Five independent experiments were performed for HSD3B1, StAR, CYP11A1 after DHA 20 μM treatment and six independent experiments for PCNA after DHA 10 μM treatment.

Cell signaling pathway assays were conducted for AMP-activated protein kinaseα (AMPKα), MAPK14, MAPK1/3 and Akt as these pathways are of interest to explore GC functions. Indeed, these pathways are involved in both cellular proliferation and steroidogenesis, the functions investigated in the present study. GCs were cultured in 48-well dishes (2.5 × 10^5^ viable cells/250 μL media/well) in modified McCoy’s 5A medium. After 15 h, 100 μL supernatant was removed and replaced with 100 μL fresh modified McCoy’s 5A medium in the presence or absence of DHA (10 or 50 μM final concentration in well) or TUG-891 (1, 10 or 50 μM final concentration in well) for 5, 10, 30 or 60 min. Four independent experiments were performed.

With all assays, analyses were performed on total protein extracts from cultured GCs or from lung tissue in the case or FFAR4 validation. Total proteins were extracted from GCs on ice in lysis buffer (10 mM Tris (pH 7.4), 150 mM NaCl, 1 mM EDTA, 2 mM EGTA, 0.5% Nonidet P40, 1% Triton X 100) containing phosphatase inhibitors (10 mM sodium fluoride, 12 mM sodium dihydrogen phosphate, 2 mM sodium orthovanadate). In the case of lung tissue, proteins were extracted on ice after 2 h lysis buffer action in order to favor membrane receptor extraction. Lysates were centrifuged for 30 min at 4 °C at 16000 g for GC proteins and 6000 g (to favor membrane receptor recovery) for lung proteins. The protein concentration in the supernatants was determined using a colorimetric assay (kit BC Assay protein quantification; Interchim, Montluçon, France) and proteins were denaturated in Laemmli buffer for 5 min at 95 °C. Protein lysates (15 μg GC or 150 μg lung) were subjected to electrophoresis on 4–12% acrylamide gel (Life technologies, Saint-Aubin, France) and electrotransferred onto 0.45-μm nitrocellulose membranes (Pall Corporation, VWR International, France). After blocking with 5% non-fat dry milk powder (NFDMP) in TBS-0.1% Tween-20 (TBST) for 90 min at room temperature, blots were incubated with appropriate primary antibodies (for final dilutions, see Table 1) in TBST with 5% NFDMP at 4 °C overnight. The membranes were then washed in TBST and incubated with the appropriate HRP-conjugated secondary antibody (final dilution 1:5000) in TBST with 5% NFDMP for 2 h at room temperature. The signal of specific bands, detected by ECL (West Dura; Thermo-Fisher Scientific, Courtaboeuf, France), was quantified using a charge-coupled device camera GeneGnome (Syngene, Cambridge, United Kingdom) with Genesys 1.5.4 software (Syngene). The analysis of signal intensity was performed using GeneTools 4.01 software (Syngene). Results for PCNA, HSD3B1, StAR and CYP11A1 expression levels are expressed as the fold change between controls and DHA treatment. Results of MAPK14, AMPKα, Akt and MAPK1/3 phosphorylation are expressed as the ratio of phosphorylated protein to total protein, normalized by the control value at the same time-point, and with time 0 min being equal to 1 (for reference).

**Table 1:** Primer sequences for real-time reverse transcription–polymerase chain reaction

Abbrev.	Gene	Accession no.	Forward primer	Reverse primer	bp	E %
*FFAR4*	Free fatty acid receptor 4	XM_865266	GGGTTCCTTTTCGATGTGAA	GCCGTGACTCTTTGGAGAAG	166	94
*GPX4*	Glutathione peroxidase 4	NM_174770	CGATACGCCGAGTGTGGTTTAC	ACAGCCGTTCTTGTCAATGAGG	261	96
*GLUT1*	Solute carrier family 2 (facilitated glucose transporter), member 1	NM_174602	CTGATCCTGGGTCGCTTCAT	ACGTACATGGGCACAAAACCA	68	113
*NFkB*	Nuclear factor of kappa light polypeptide gene enhancer in B-cells 1	NM_001076409.1	GCACCACTTATGACGGGACT	CCATGTCCAGAGGAGTGGTT	195	89
*PPARA*	Peroxisome proliferator-activated receptor alpha	NM_001034036.1	CCTACGGGAATGGCTTCATA	GCACAATACCCTCCTGCATT	219	97
*PPARG*	Peroxisome proliferator-activated receptor gamma	Y12419/Y12420	CCCTGGCAAAGCATTTGTAT	ACTGACACCCCTGGAAGATG	222	88
*RPL19*	Ribosomal protein L19	BC102223	AATCGCCAATGCCAACTC	CCCTTTCGCTTACCTATACC	156	94
*RPS9*	Ribosomal protein S9	BC148016	GGAGACCCTTCGAGAAGTCC	GGGCATTACCTTCGAACAGA	180	100
*SREBF1*	Sterol regulatory element binding transcription factor 1	AB355703.1	ACCGCTCTTCCATCAATGAC	TTCAGCGATTTGCTTTTGTG	190	97

*Abbrev* gene abbreviation, *bp* product size in base pair, *E* primer efficiency

### Gene expression analysis in GCs

GCs were cultured in 48-well dishes (2.5 × 10^5^ viable cells/250 μL media/well) in modified McCoy’s 5A medium in the presence or absence of DHA (1, 10, 20 or 50 μM) or TUG-891 (1, 10 or 50 μM) for 8 h. After removal of the medium, cells were recovered using 200 μl/well of TriZol reagent (Invitrogen, Cergy Pontoise, France), immediately frozen and stored until analysis. An additional condition, consisting in GC collected from ovaries and then immediately frozen and stored until analysis was also constituted and named in vivo GC. Total RNA was extracted from GCs according to the manufacturer’s instructions. RNA concentration was determined using a NanoDrop ND-1000 spectrophotometer (Nyxor Biotech, Paris, France). DNAse treatment and reverse transcription (RT) was performed on 1 μg of total RNA extracted from GCs using Maxima First Strand cDNA Synthesis kit (Thermo-Fisher Scientific) according to the manufacturer’s recommendations. Real-time PCR reactions were carried out on a CFX96 (Bio-Rad, Marnes-la-Coquette, France) in 20 μL volumes containing primers, each at a final concentration of 150 nM (Table 1), 5 μL of the diluted RT reaction (10 ng cDNA per reaction) and qPCR Mastermix Plus for Sybr Green I (Bio-Rad) according to the manufacturer’s instructions. As expression of *FFAR4* and *FFAR1* in ovarian tissue is low, real-time RT-PCR were performed on 50 ng cDNA per reaction. The efficiency of the primers (Table 2) and standard curve for each gene were calculated from serial dilutions of the corresponding cDNA fragment obtained as a template. Relative gene expression levels were determined in ten independent GC samples for each treatment. The geometric mean of two housekeeping genes, Ribosomal protein L19 (*RPL19*) and Ribosomal protein S9 (*RPS9*), was used to normalize gene expression. The relative amounts of gene transcripts (R) were calculated according to the equation: $\mathrm{R}=\frac{\left({E}_{gene}^{- Ct\  gene}\right)}{\left(\mathrm{geometric}\ \mathrm{mean}\ \left({E}_{RPS9}^{- Ct\  RPS9};{E}_{RPL19}^{- Ct\  RPL19}\right)\right)\ }$, where E is the primer efficiency and Ct the cycle threshold.

**Table 2:** Fatty acid composition from total lipids of bovine granulosa cells after 15 h treatment with DHA

Fatty acids (mole % of total fatty acids)	Control ^a^	DHA1 μM	DHA 10 μM	DHA 50 μM
	*n* = 5	*n* = 4	*n* = 4	*n* = 3
Saturates				
16:0	31.50 ± 7.48	40.16 ± 9.38	44.12 ± 11.60	48.45 ± 7.44
18:0	10.68 ± 2.25	13.36 ± 3.03	15.04 ± 3.89	16.24 ± 2.60
Monounsaturates				
16:1 n-7	2.32 ± 0.55	3.03 ± 0.72	3.33 ± 0.91	3.52 ± 0.68
18:1 n-7	8.93 ± 2.17	11.51 ± 2.85	12.36 ± 3.72	12.22 ± 3.01
18:1 n-9 cis	29.99 ± 7.18	38.91 ± 9.08	42.49 ± 11.58	43.03 ± 7.86
n-6 PUFA				
18:2 n-6 cis	6.05 ± 1.29	7.87 ± 1.81	8.59 ± 2.33	8.41 ± 2.07
20:4 n-6	8.85 ± 2.12	11.87 ± 3.08	13.02 ± 3.99	11.24 ± 4.10
n-3 PUFA				
18:3 n-3	0.51 ± 0.12	0.66 ± 0.15	0.74 ± 0.18	0.67 ± 0.17
20:5 n-3	0.98 ± 0.25	1.34 ± 0.31	1.60 ± 0.38	1.37 ± 0.42
22:5 n-3	7.03 ± 1.72	9.33 ± 2.28	10.04 ± 2.90	7.79 ± 3.31
22:6 n-3 (DHA)	0.84 ± 0.16	2.17 ± 0.04 *	10.27 ± 0.11 *	23.05 ± 6.58 *

Granulosa cells (GC) (2.5 × 10^5^ viable cells/250  μL media/well) were cultured into 48-well dishes in modified McCoy’s 5A media in the presence or absence of DHA (1, 10 or 50 μM). After 15  h culture, supernatants were removed and cells with the same treatment (12 wells) were pooled and stored under nitrogen gas at −80 °C until the total lipid extraction. The fatty acid analysis was performed by gas chromatography by the Plateforme de Lipidomique Fonctionnelle (INSA, Villeurbanne, France). The relative amount of each fatty acid is expressed as the area of each fatty acid peak, relative to the total area for all fatty acids (mole%). The results are expressed as means ± SEM (*n* = 4 per treatment). ^a^ Control corresponds to the serum-free modified McCoy’s 5A medium supplemented with DMSO (1:2000) as carrier solvent of DHA. The prepared mediums with the different concentrations of DHA were all adjusted for DMSO at 1:2000. ^*^ indicates a significant difference with control condition (Kruskal-Wallis test with Tukey’s multiple comparison test as post-hoc test, *p* < 0.05)

### Statistical analysis

Statistical analyses were performed for GC proliferation, steroidogenesis, gene expression, protein expression, GC lipid composition taking into account treatment effect and replica effect. Concerning cell signaling, the time effect and replica effect were analyzed. GC proliferation, progesterone secretion, estradiol secretion, GC lipid composition and cell signaling were compared between the groups. A one-way ANOVA was used when distribution and variance enabled performance of a parametric study (Shapiro test, Levene test, Rcmdr package), with Tukey’s post hoc comparison (R package multcomp [46]). A non-parametric ANOVA (permutational ANOVA) was used when distribution was not normal and variance was not homogenous (R package lmPerm [47]), with a Tukey’s post-hoc test (R package nparcomp [48]), R version 3.3.1 [49]). Gene expression was analyzed using K-sample Fisher Pitman permutation test with a Monte Carlo approximation (Rcmdr package) with the treatment as a fixed factor and the replica as a random factor, with Tukey’s post hoc comparison (R package nparcomp). Protein expression levels of PCNA, HSD3B1, StAR and CYP11A1 were analyzed using the Kruskal-Wallis test (Shapiro test, Levene test, Rcmdr package). A *p*-value of ≤0.05 was considered to indicate a significant difference and 0.05 < *p* ≤ 0.10 a tendency.

## Results

### FFAR4 expression

**Fig. 1:**
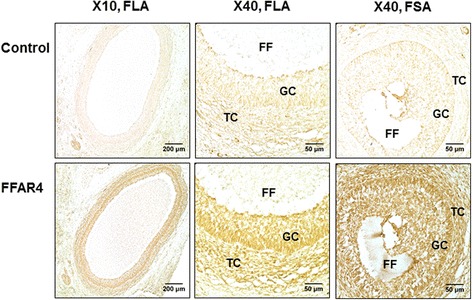
Expression of free fatty acid receptor 4 (FFAR4) in bovine ovarian follicles by immunohistochemistry. Immunohistochemistry was performed on sections of ovarian follicles. FFAR4 (brown labeling) was immunodetected in ovarian follicles with large (FLA) or small (FSA) antrum (customized FFAR4 rabbit antibody, Agro-Bio). Pre-immunized rabbit serum was used as the control with the same secondary antibody as for FFAR4 detection. Bars = 200 μm and 50 μm for 10× and 40× microscope objectives, respectively. FF - follicular fluid, GC – granulosa cells, TC – theca cells

**Fig. 2:**
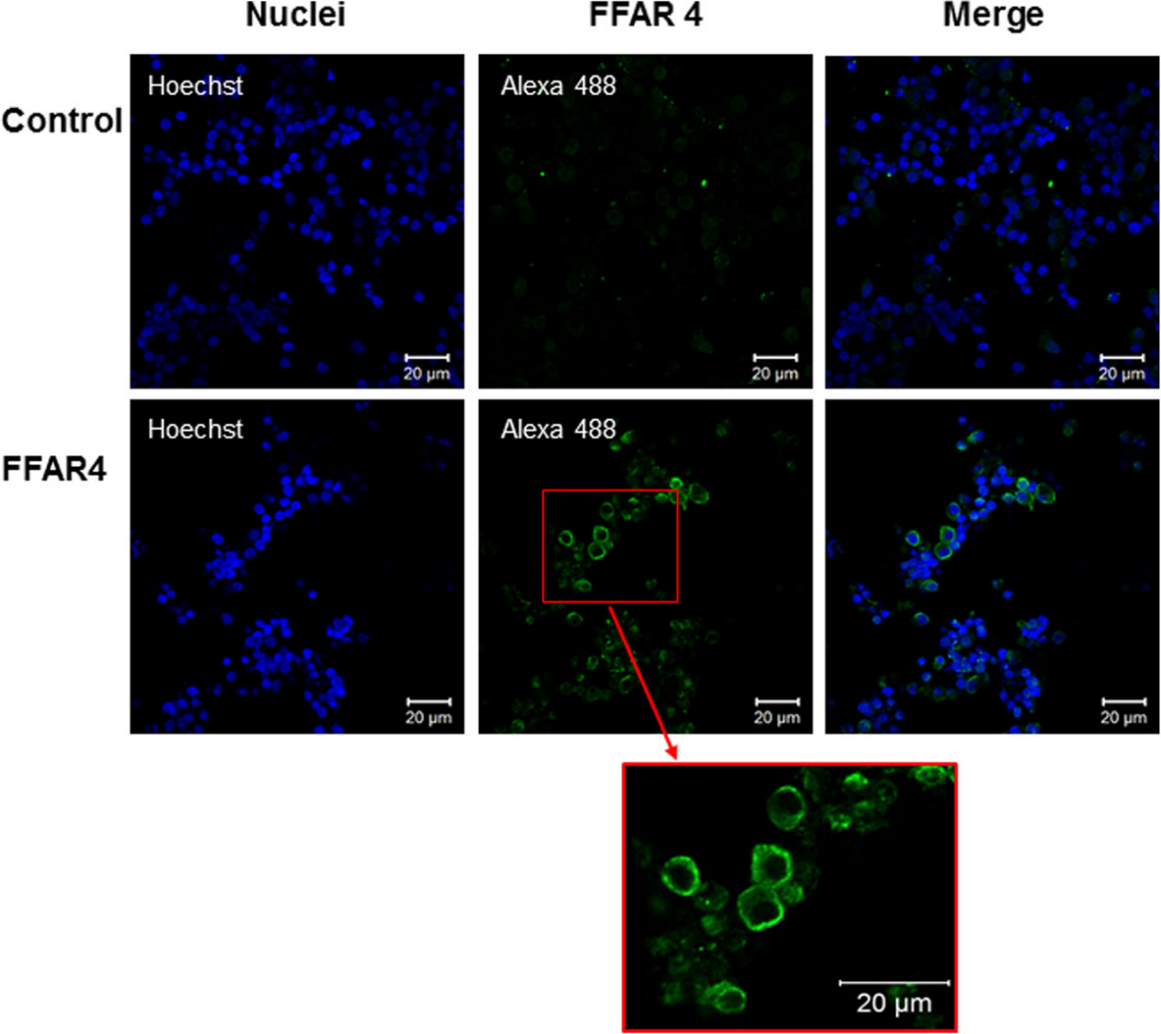
Expression and localization of free fatty acid receptor 4 (FFAR4) in bovine granulosa cells by immunofluorescence. Immunofluorescence was performed on granulosa cells (GCs) after in vitro culture. Briefly, recovered GCs after follicle puncture and GC washing were incubated in serum-free modified McCoy’s 5A medium (2.5 × 10^5^ viable cells/well on a 8-well chamber slide (Lab-Tek® Nunc) for 48 h. Cultures were performed in a water-saturated atmosphere containing 5% CO_2_ in air at 38 °C. FFAR4 (green fluorescence) was immunodetected in GCs (customized FFAR4 rabbit antibody, Agro-Bio). Pre-immunized rabbit serum was used as the control with the same secondary antibody as for FFAR4 detection. Nuclei were stained with Hoechst 33,258 (blue fluorescence). The picture framed in red is a magnification of the area framed in red from the original image. Bars = 20 μm

Both *FFAR1* and *FFAR4* mRNA were detectable at a low level in several ovarian compartments, such as the cortex and theca, granulosa and cumulus cells (Additional file 4: Figure S3). An antibody was designed against a peptide from the second extracellular loop of the FFAR4 bovine protein. The signal corresponding to the 42 kDa band was extinguished by increasing the quantity of peptide used to prepare the antibody, demonstrating that the band corresponded to FFAR4 (Additional file 1: Figure S1). The protein FFAR4 was detected by both immunohistochemistry (brown labelling, Fig. 1) on bovine ovarian follicles and immunofluorescence (green fluorescence, Fig. 2) on cultured bovine GCs, using the bovine FFAR4 antibody. FFAR4 protein was present in all cellular types of follicles with small and large antrum. FFAR4 protein immunofluorescence seemed to exhibit a peripheral labelling close to the cellular membrane in some GCs and the intensity and presence of the staining was variable in GCs cultured in vitro (Fig. 2). The precise localization of the labelling to the cellular membrane is not obvious in all cells. This signal is similar to FFAR4 immunofluorescence in bovine GCs, obtained using a commercial antibody design against a peptide of the human protein sharing 89% identity with the bovine FFAR4 sequence (Additional file 5: Figure S4).

### Cellular proliferation

**Fig. 3:**
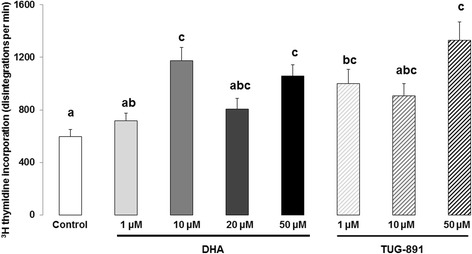
Synthesis of DNA in bovine granulosa cells: ^3^H-thymidine incorporation after 24 h treatment with DHA or TUG-891. Effects of DHA or TUG-891 on cell proliferation were assessed by measurement of ^3^H-thymidine incorporation in bovine granulosa cells after 24 h culture in enriched McCoy’s 5A media with various doses of DHA (1, 10, 20 and 50 μM) or TUG-891 (1, 10 and 50 μM), as described in Material and Method section. The chemical DMSO alone (1/2000) was used as a negative control due to its use as a solvent for DHA and TUG-891. The data are expressed as disintegrations per minute. Results represent 13 independent cultures with each treatment conducted in four replicates and are presented as mean ± SEM. Bars with different superscripts are significantly different (*p* < 0.05)

DHA significantly increased basal cellular proliferation at 10 μM (1.9-fold increase; *p* < 0.0001) and 50 μM (1.8-fold increase; *p* < 0.0001) compared to the control (Fig. 3). A similar increase in GC proliferation was also observed with TUG-891 at 1 μM (1.7-fold increase; *p* = 0.014) and 50 μM (2.2-fold increase; *p* < 0.0001; Fig. 3).

### Steroidogenesis

**Fig. 4:**
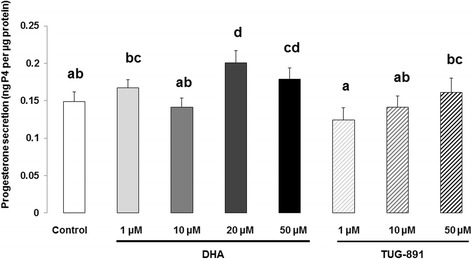
Progesterone secretion from bovine granulosa cells after 48 h treatment with DHA or TUG-891. Effects of DHA or TUG-891 on progesterone secretion were assessed in culture media of bovine granulosa cells cultured for 48 h in enriched McCoy’s 5A media with various doses of DHA (1, 10, 20 and 50 μM) or TUG-891 (1, 10 and 50 μM), as described in Material and Method section. The chemical DMSO alone (1/2000) was used as a negative control due to its use as a solvent for DHA and TUG-891. Progesterone secretion was normalized with the protein concentration in each well and expressed as ng progesterone (P4) per μg protein. Results of 12 independent cultures, with each treatment conducted in quadruplicate, are presented as mean ± SEM. Bars with different superscripts are significantly different (*p* < 0.05)

DHA significantly increased basal progesterone secretion at 20 μM (1.3-fold increase; *p* < 0.0001) and 50 μM (1.2-fold increase; *p* = 0.028) compared to the control (Fig. 4). With regard to the effect of TUG-891, no significant effect was observed on progesterone secretion at any concentration (Fig. 4).

**Fig. 5:**
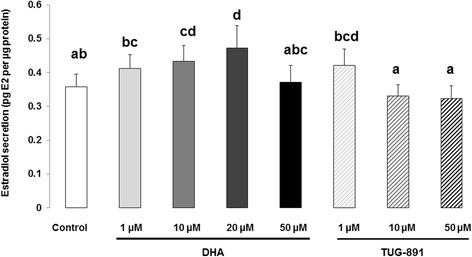
Estradiol secretion from bovine granulosa cells after 48 h treatment with DHA or TUG-891. Effects of DHA or TUG-891 on estradiol secretion were assessed in culture media of bovine granulosa cells cultured for 48 h in enriched McCoy’s 5A media with various doses of DHA (1, 10, 20 and 50 μM) or TUG-891 (1, 10 and 50 μM), as described in Material and Method section. The chemical DMSO alone (1/2000) was used as a negative control due to its use as a solvent for DHA and TUG-891. Estradiol secretion was normalized with the protein concentration in each well and expressed as pg estradiol (E2) per μg protein. Results of six independent cultures, with each treatment conducted in quadruplicate, are presented as mean ± SEM. Bars with different superscripts are significantly different (*p* < 0.05)

DHA significantly increased basal estradiol secretion at 10 μM (1.2-fold increase; *p* = 0.030) and 20 μM (1.3-fold increase; *p* < 0.0001) compared to the control (Fig. 5). No effect of TUG-891 on estradiol secretion was reported at any concentration (Fig. 5).

### Protein expression

**Fig. 6:**
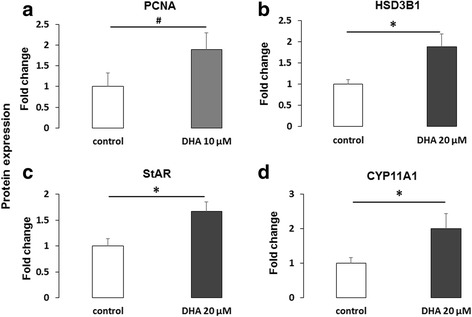
Protein expression of (**a**) proliferating cell nuclear antigen (PCNA), (**b**) hydroxy-delta-5-steroid dehydrogenase, 3 beta- and steroid delta-isomerase 1 (HSD3B1), (**c**) steroidogenic acute regulatory protein (StAR) and (**d**) cytochrome P450 family 11 subfamily A member 1 (CYP11A1) after 15 h treatment with DHA. Effects of DHA treatment on protein levels were assessed in bovine granulosa cells after 15 h culture in enriched McCoy’s 5A media in presence or absence of DHA 10 or 20 μM. The chemical DMSO alone (1/2000) was used as a negative control due to its solvent activity on DHA. Protein extracts were separated by electrophoresis on 4–12% (w:v) SDS-polyacrylamide gel. After electrotransfer to nitrocellulose membranes, the proteins were probed with anti-PCNA (**a**), anti-HSD3B1 (**b**), anti-StAR (**c**) or anti-CYP11A1 (**d**) antibodies. The blots were stripped and re-probed with antibodies against Vinculin (VCL). Results of at least five independent experiments are presented. Bands on the blots were quantified and the total protein / VCL protein ratio was calculated. Results are expressed relative to the control as mean ± SEM of five independent experiments for HSD3B1, StAR, CYP11A1 and six independent experiments for PCNA. * indicates significant difference (*p* < 0.05) and # indicates tendency (*p* < 0.10)

PCNA expression level was analyzed by western blotting in GCs after 15 h in the presence or absence of DHA (10 μM; Fig. 6a Additional file 6: Figure S6), as this dose exhibited the highest significant effect on GC proliferation. DHA showed a tendency to increase PCNA expression levels (1.9-fold increase; *p* = 0.055). Expression levels of the steroidogenic enzymes, HSD3B1 and CYP11A1, and the cholesterol transporter StAR, were analyzed by western blotting in GCs after 15 h in the presence or absence of DHA (20 μM; Fig. 6b-d, respectively, Additional file 6: Figure S6), as this dose exhibited the highest significant effect on GC progesterone and estradiol secretion. DHA showed a significant 1.88-fold increase in HSD3B1 expression (*p* = 0.028), a significant 1.67-fold increase in StAR expression (*p* = 0.016) and a significant 2-fold increase in CYP11A1 expression (*p* = 0.047).

### Gene expression

**Fig. 7:**
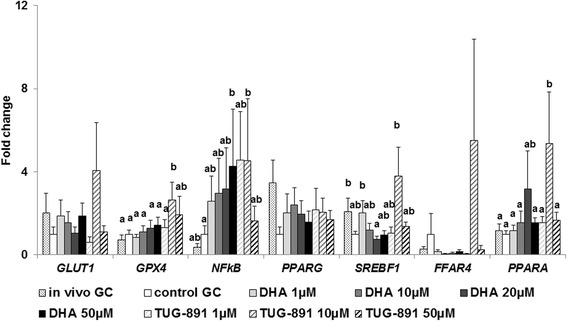
Gene expression in bovine granulosa cells before or after 8 h treatment with DHA or TUG-891. Effects of DHA or TUG-891 on mRNA expression of solute carrier family 2 member 1 (*GLUT1*), glutathione peroxidase 4 (*GPX4*), nuclear factor of kappa light polypeptide gene enhancer in B-cells 1 (*NFkB*), peroxisome proliferator-activated receptor gamma (*PPARG*), sterol regulatory element binding transcription factor 1 (*SREBF1*), free fatty acid receptor 4 (*FFAR4*) and *PPAR* alpha (*PPARA*) were assessed in bovine granulosa cells (GC) before or after 8 h culture in enriched McCoy’s 5A media with various doses of DHA (1, 10, 20 and 50 μM) or TUG-891 (1, 10 and 50 μM), as described in Material and Method section. The chemical DMSO alone (1/2000) was used as a negative control due to its use as a solvent for DHA and TUG-891. Total mRNA was extracted from GC and reverse-transcribed, and real-time RT-PCR was performed. The geometric mean of two housekeeping genes (*RPL19*- ribosomal protein L19 and *RPS9*- ribosomal protein S9) was used to normalize gene expression. Results of 10 independent cultures are presented as mean ± SEM. Bars with different superscripts are significantly different (*p* < 0.05)

Gene expression of several candidate genes previously reported to be involved in n-3 PUFA effects: *GLUT1* (involved in glucose metabolism), *GPX4* (involved in oxidative stress), *PPARA*, *PPARG* and *SREBF1*, 3 transcription factors (involved in lipid metabolism) and *FFAR4* (membrane receptor able to bind DHA and *NFκB*, a signaling molecule reported to be involved in DHA action) (Fig. 7). Four genes showed a significant condition effect: *GPX4* (*p* = 0.026), *NFκB* (*p* = 0.003), *PPARA* (*p* = 0.050) and *SREBF1* (*p* = 0.015). *GPX4*, *PPARA* and *NFκB* exhibited a significant 2.6-fold increase (*p* = 0.004), 5.4-fold increase (*p* = 0.004), and 4.5-fold increase (*p* = 0.018), respectively, in expression with TUG-891 (10 μM) compared to the control. NFκB also exhibited a significant 4.3-fold increase in expression with DHA 50 μM compared to the control (*p* = 0.028). No other treatments caused any difference in gene expression compared to controls.

### Cell signaling pathways

**Fig. 8:**
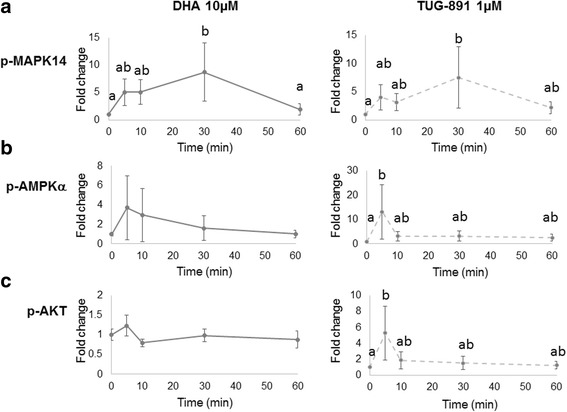
Signaling pathways in bovine granulosa cells after DHA or TUG-891 treatment. Effects of DHA or TUG-891 on phosphorylation of (**a**) mitogen-activated protein kinase 14 (MAPK14), (**b**) AMP-activated protein kinaseα (AMPKα) and (**c**) protein kinase B (Akt) signaling pathways were assessed in bovine granulosa cells cultured for 15 h in enriched McCoy’s 5A media with 10 μM DHA or 1 **μ**M TUG-891, as described in Material and Method section for 5, 10, 30 and 60 min. Protein extracts were separated by electrophoresis on 4–12% (w:v) SDS-polyacrylamide gel. After electrotransfer to nitrocellulose membranes, the proteins were probed with anti-phosphorylated (p-)MAPK14 (**a**), anti-p-AMPKα (**b**) or anti-p-AKT1/2/3 (**c**) antibodies. The blots were stripped and re-probed with antibodies against MAPK14, AMPKα or Akt, respectively. Bands on the blots were quantified. Results of four independent experiments are presented as the ratio of p-protein to total protein, normalized by the ratio observed in control at each time and expressed as mean ± SEM of four independent experiments, with time 0 min being equal to 1 (for reference). Bars with different superscripts are significantly different (*p* < 0.05)

MAPK14, AMPKα, Akt and MAPK1/3 pathways were investigated after 5, 10, 30 or 60 min in the presence or absence of DHA (10 or 50 μM) or TUG-891 (1, 10 or 50 μM, Fig. 8, Additional file 7: Figure S5 and Additional file 8: Figure S7). The non parametric ANOVA showed a significant treatment effect for Akt (*p* = 0.005), AMPKα (*p* = 0.001), MAPK1/3 (*p* = 0.048) and MAPK14 (*p* < 0.001). Only for MAPK14 phosphorylation, a significant time effect was reported (*p* < 0.001), and significant time by treatment interaction were reported for MAPK14 (*p* < 0.0001), MAPK1/3 (*p* = 0.040) and AMPKα (*p* = 0.008). The significant time by treatment interaction meant that the cells did not respond similarly across time to all doses of DHA and TUG-891. We therefore analyzed the effect of time for each treatment used.

The main changes were observed on MAPK14 pathway (Fig. 8a and Additional file 7: Figure S5A). Treatment with DHA (10 μM) led to a significant 8.7-fold increase in MAPK14 phosphorylation at 30 min compared to the control (*p* = 0.007; Fig. 8a). Treatment with DHA (50 μM) led to significant 5.4-fold and 8.4-fold increases in MAPK14 phosphorylation at 5 min (*p* = 0.035) and 30 min (*p* = 0.001), respectively; a similar tendency was observed at 10 min (5.0-fold increase; *p* = 0.054, Additional file 5: Figure S4A). Treatment with TUG-891 (1 μM and 50 μM) led to significant 7.5-fold and 22.5-fold increases in MAPK14 phosphorylation at 30 min, respectively, compared to the control (*p* = 0.026 and 0.024, respectively; Fig. 8a; Additional file 5: Figure S4A). Treatment with TUG-891 (10 μM) led to a significant 7.6-fold increase in MAPK14 phosphorylation at 10 min compared to the control (*p* = 0.031); a similar tendency was observed at 5 min (6.0-fold increase; *p* = 0.091; Additional file 5: Figure S4A). Whilst no effect was observed on Akt and AMPKα phosphorylation after DHA treatment at 10 μM (Fig. 8c) or 50 μM (data not shown), treatment with TUG-891 (1 μM) resulted in a significant 5.3-fold increase in Akt phosphorylation at 5 min (*p* = 0.020; Fig. 8c) and a 13.2-fold increase in AMPKα phosphorylation at 5 min (*p* = 0.029; Fig. 8b) compared to the control. Moreover, treatment with TUG-891 (50 μM) led to a significant 5.8-fold increase in AMPKα phosphorylation at 30 min (*p* = 0.039); a similar tendency was observed at 60 min (5.5-fold increase; *p* = 0.053; Additional file 7: Figure S5B). Finally, no effect was observed on MAPK1/3 phosphorylation with DHA (10 or 50 μM) or TUG-891 (1 or 10 μM) (data not shown). Only a transient, albeit significant, increase in MAPK1/3 phosphorylation was observed after 5 min treatment with TUG-891 at 50 μM (1.6-fold increase; *p* = 0.021; Additional file 7: Figure S5C).

### DHA incorporation in granulosa cells

Fatty acid composition was analysed in GC after DHA treatment on total fatty acids (Table 2). DHA 1 μM led to a significant 2.6-increase (*p* < 0.032) 10 μM led to a significant 12-fold increase (*p* < 0.0055) and DHA 50 μM led to a significant 27-fold increase (*p* < 0.0016) in DHA lipid composition. No other difference in FA lipid composition was reported between groups.

## Discussion

In the present study, we investigated the effect of DHA treatment on bovine GCs in culture. For the first time, we showed that the addition of 10 μM and 50 μM DHA increased cellular proliferation, that the addition of 20 μM and 50 μM DHA increased progesterone secretion and that the addition of 10 μM and 20 μM DHA increased estradiol secretion. We also performed experiments to decipher the possible mechanisms of DHA action on GC functions. We showed that DHA increased PCNA, StAR and steroidogenic enzyme HSD3B1 and CYP11A1 protein expression and MAPK14 phosphorylation. We also reported, for the first time, the mRNA and protein expression of FFAR4 in bovine GCs and showed that FFAR4 activation by the FFAR4 agonist TUG-891 led to a similar increase in cellular proliferation and MAPK14 phosphorylation as with DHA treatment. However, TUG-891 treatment did not lead to increases in steroid secretion.

### DHA increased in vitro granulosa cell proliferation

We reported here that DHA (10 and 50 μM) increased cellular proliferation of GCs in vitro. This finding is relevant to the increase in PCNA expression that we also observed after DHA treatment. Indeed, PCNA is involved in both DNA synthesis and repair [50]. Such effects of n-3 PUFAs on GC proliferation have already been reported in ovine cells in vitro [51]. Moreover, our in vitro results are also consistent with in vivo n-3 PUFA dietary supplementation data previously reported for bovine animals. Indeed, n-3 PUFA-enriched diets have resulted in increases in the pre-ovulatory follicle size [9, 10], and the number of total follicles [16], small follicles [17], medium follicles [52, 53] and large follicles [11] in bovine animals. These data are consistent with a stimulatory effect of n-3 PUFAs on GC proliferation, which is the crucial step involved in the increase in size of the entire follicle. Indeed, an increase in follicle numbers corresponds to increase in follicles that are detectable by ultrasonographic examination (> 2–3 mm). The increase of follicles of this size is a consequence of follicles < 2 mm growth and of the proliferation of their GCs.

### DHA increased in vitro granulosa cell steroidogenesis

With regard to steroidogenesis, we reported here that DHA increased secretion of both progesterone and estradiol. This finding is consistent with increases in the protein expression level of the steroidogenic enzymes, HSD3B1, CYP11A1 and of the carrier StAR that we observed. Indeed, StAR protein is essential for the initial and rate-limiting step in steroid biosynthesis, namely transfer of hydrophobic cholesterol from the outer mitochondrial membrane to the inner mitochondrial membrane (reviewed in [54]), the location of another enzyme crucial to steroid biosynthesis, CYP11A1. This enzyme converts cholesterol into pregnenolone [55]. The HSD3B1 enzyme is a required step in the conversion of pregnenolone into progesterone [56] or in theca cells to further obtain androstenedione [57], the latter being a necessary precursor in estradiol synthesis. Moreover, such stimulating effects of n-3 PUFAs on steroidogenesis have already been described for ovine GCs in vitro, involving both progesterone and estradiol secretion [51]. The increase in progesterone and estradiol observed after DHA treatment is also consistent with our previous results, which showed a stimulatory effect of DHA on progesterone secretion from bovine cumulus cells, originating from GCs [19]. N-3 PUFAs have also been shown to increase progesterone secretion from ovine theca cells in vitro [58] and follicular fluid progesterone concentration (from follicles 4–7 mm diameter) in vivo, in ewes fed with a n-3 PUFA-enriched diet [51]. Furthermore, in agreement with the increase in StAR protein level measured in the present study, Waters et al. previously showed that a n-3 PUFA-enriched diet led to increased *StAR* gene expression in another bovine reproductive compartment, the endometrium [59]. In mouse Leydig tumor cells, inhibition of PTGS2 activity (an enzyme required for prostaglandin biosynthesis) is known to facilitate cAMP-induced steroidogenesis via increased StAR protein expression [60]. N-3 PUFAs are known to be potent inhibitors of PTGS2 activity [61]; this may explain the increase in StAR protein expression reported in the present study, following DHA treatment of GCs. The increased estradiol effect observed after DHA treatment indicates that DHA might also affect cytochrome P450 aromatase and/or 17β-HSD protein expression levels, as both of these enzymes are necessary for conversion of androstenedione (supplied in our culture media) to estradiol [62]. However, this hypothesis would require further investigation. Finally, examination of steroidogenic enzyme activities would clarify whether DHA affects expression levels only or also the activities of these enzymes.

### DHA mechanisms of action through FFAR4

For the first time, we showed in the present study that FFAR4 is expressed in bovine granulosa cells. To our knowledge, granulosa expression of FFAR4 has not yet been reported in other species. Only a few published papers have reported FFAR4 expression in bovine tissues, namely adipose tissue [63, 64]. In other species, FFAR4 is expressed in adipose tissue, spleen and intestine in pigs [65], while in human and rodents, its expression is more widely reported in additional tissues (immune cells, pancreatic cells, lung, muscle, liver and placenta) [42, 66–69]. We demonstrated here that FFAR4 is localized in the vicinity of the cellular membrane in GCs, as expected for a G protein-coupled receptor. This expression of FFAR4 in bovine GCs indicates that long chain fatty acids, including DHA, could act on GCs, at least in part, via this receptor. However, it should be noted that not all cultured GCs were shown to express FFAR4.

Activation of FFAR4 by TUG-891 (1 and 50 μM) mirrored the effect of DHA on GC proliferation, but not on progesterone and estradiol secretion, suggesting that DHA effects on cell proliferation could occur through FFAR4 activation. Indeed, TUG-891 is a specific and potent agonist of FFAR4 [41, 70]. Thus, the effects observed after TUG-891 treatment could be related to FFAR4 activation. It has already been reported that DHA could exert effects through FFAR4 stimulation in adipocytes [71], in macrophages [72] or in cardia cells [73]. Of note, a linear dose response was not reported in the present study, notably concerning GC proliferation (DHA 20 μM). This observation could suggest that the DHA dose response followed a U-shaped (for example) response pattern [74]. It is also possible that, at higher concentration, other DHA mechanisms could occur, potentially counteracting the FFAR4 effect on proliferation.

Moreover, several effects, such as the ability of DHA to inhibit responsiveness of macrophages to endotoxin, inhibition of IκB kinase phosphorylation, IκB phosphorylation and degradation, and inhibition of production of TNF, IL-6 and MCP-1, that have already been demonstrated, were abolished in FFAR4 knockdown cells [28, 75]. In our study, we observed an increase in MAPK14 phosphorylation in GCs after DHA supplementation (10 and 50 μM), and a similar increase after TUG-891 treatment (1, 10 and 50 μM). As shown in the published literature, MAPK14 (p38 MAPK) may be involved in follicle stimulating hormone (FSH)-induced estradiol secretion in rat and mouse GCs [37, 38], but was also demonstrated to be involved in GC proliferation in a culture model of hamster pre-antral follicles stimulated with transforming growth factor β1 (TGFβ1) [76]. Thus, these results suggest that FFAR4 activation leads to the activation of this MAPK14 signaling pathway, which might play a role in DHA effects on bovine GC proliferation. However, additional experiments would be needed to demonstrate that DHA could activate FFAR4 in bovine GCs, involving investigation of, for example, plasmid transfection, FFAR4 overexpression and inhibition, and intracellular calcium measurement.

No effect of TUG-891 treatment was reported on GC steroidogenesis, suggesting that DHA effects on progesterone and estradiol secretion are independent of FFAR4 activation. Several other mechanisms of action have already been proposed for DHA: modification of cellular membrane lipid composition (fluidity, raft formation); regulation of eicosanoid production (for example, prostaglandins and leukotrienes); transcription factor expression (*PPARG* and *SREBF1,* for example) or signaling pathways, such as NFκB [26, 28, 30]. In this study, we evidenced a significant increase in cellular DHA level from total lipids after 1 μM, 10 μM and 50 μM DHA treatment. It is thus possible that DHA affected steroidogenesis by one of these mechanisms and not by activating FFAR4.

The study of candidate gene expression that was performed here showed no modification of canonical genes (*PPARG*, *PPARA*, *SREBF1*) following DHA treatment, except for *PPARA* with the highest DHA dose used, DHA50 μM. Similar absence of effects on *PPARG* have already been reported in other studies, as in this study [77]. Nevertheless, TUG-891 treatment has already been reported to modify *PPARG* transcription after several days of culture in 3T3L1 [71]. In this study, we analyzed GC gene expression after 8 h of TUG-891 stimulation, which might be too brief a period to enable transcription modifications to occur immediately after FFAR4 stimulation. The only differences observed in gene expression are upregulations of *GPX4* (glutathione peroxidase 4), *NFκB* (nuclear factor kappa B) and *PPARA* (peroxisome proliferator-activated receptor alpha) expression after TUG-891 treatment (10 μM). GPX4 is a phospholipid hydroperoxidase that protects cells against membrane lipid peroxidation. Such a mechanism has already been described for DHA in murine hippocampal cells [78]. By increasing *GPX4* expression, DHA is able to protect the cell from oxidative damage resulting of non-enzymatic peroxidation of membrane phospholipids. It is possible that this mechanism is associated with FFAR4 activation, as we found a similar increase in *GPX4* expression after TUG-891 treatment. DHA is able to bind *PPARG* and *PPARA* and can consequently increase insulin sensitivity [26]. Through binding to *PPARG*, DHA could inhibit activation of NFκB, a key transcription factor involved in inflammatory pathways [26]. Moreover, the inhibitory effect of DHA on *NFkB* can also occur via the FA receptor FFAR4 [28]. The increased *NFkB* mRNA expression reported after DHA 50 μM and TUG 10 μM in the present study is thus surprising and NFκB activation, which was not investigated in the present study, should be studied.

Overall, despite functional differences between DHA-treated and control GCs, there were no huge changes in gene expression. In order to further investigate the effects and mechanisms of action of FFAR4 activation, experiments involving primary GC culture should be replaced by those involving GC line cultures; such cultures would enable FFAR4 overexpression and inactivation, while maintaining a GC phenotype. It is also possible that a global transcriptomic approach should be envisioned after DHA treatment in order to have a broad picture of the potential mechanisms involved. Indeed, the investigation of other genes, such as PTGS2, might have shown differences that could explain StAR regulation, for example.

Of note, the culture system used in this experiment is serum free and prevent the differentiation of granulosa cells generally occurring after about 15 h culture. It also enable to treat cells with the precise concentrations of DHA (which would be already present in culture medium if we had used serum). In the culture system used in the present paper, GC are maintaining a round shape even when platted, at least till 48 h culture (the latest endpoints in this paper), and not a fibroblastic-like shape, meaning they did not differentiate as much as when serum is used. On the other hand, this culture system also presented some disadvantages. Indeed, primary cell cultures require freshly isolating GCs for each culture, and therefore they can exhibit huge variation in response to treatment, depending on the batch of ovaries used. In this context, the use of a high number of independent culture can be compulsory, as in the present work. Moreover, in this culture system, in order to be able to set up endpoints before 48 h culture, we chose to treat cells at the beginning of the culture, meaning that both platted and non-platted cells were treated. A similar number of living cells is cultured in each well, with a varying proportion of dead cells (assessed by trypan blue staining and cell counting). Concerning proliferation assay, as floating cells are removed before the assay, only thymidine incorporated in platted cells is measured (most of floating cells being dead cells in our cell culture system). Concerning data on steroidogenesis, steroid secreted in the culture medium by both platted and non-platted cells during the cell culture are measured after 48 h. Steroid concentration are normalized by protein concentration in each well, meaning by protein amount of platted cells only. We estimated that the proportion of platted cells to viable floating cells is the same in each well for a specific batch of cells. This normalization might biased the steroid results as the concentration of steroid for a viable amount of cells might be slightly overestimated due to the normalization taking into account only platted cells. Nevertheless, we believe that this normalization would not affect differences observed between conditions.

## Conclusions

The present study reported that DHA treatment during in vitro culture increased granulosa cell proliferation and PCNA expression level, suggesting an effect of this n-3 PUFA on DNA synthesis. This effect is relevant with the increase in ovarian follicular population observed after in vivo n-3 enriched diet supplementation of dairy cows. DHA supplementation also increased progesterone and estradiol secretion, together with the protein expression level of the steroidogenic enzymes HSD3B1, CYP11A1 and the cholesterol transporter StAR. Such increase in progesterone level is also reported after in vivo n-3 enriched diet supplementation of dairy cows. These effects on granulosa cell function could thus be related to the improved reproduction observed after n-3 enriched diet supplementation. TUG-891, a FFAR4 agonist, showed similar effects to DHA on GC proliferation and on MAPK14 phosphorylation, but had no effect on steroidogenesis. These data indicate that DHA might act on GC proliferation through FFAR4 activation, which, in turn, leads to MAPK14 phosphorylation. Nevertheless, FFAR4 activation by DHA remains to be demonstrated in bovine GCs. Other potential mechanisms of DHA action on steroidogenesis should be investigated, as our hypothesis of FFAR4-mediated effects on steroidogenesis was not verified.

## Additional files

Additional file 1: **Figure S1.** Control of customized free fatty acid receptor 4 (FFAR4) antibody specificity. Protein extracts from bovine lung tissue were separated by electrophoresis on 4–12% (w:v) SDS-polyacrylamide gel. After electrotransfer to nitrocellulose membranes, the proteins were probed with anti-FFAR4 antibody (0.95 μg/mL, customized FFAR4 rabbit antibody, Agro-Bio), which was pre-incubated for 15 min with different concentrations of the bovine specific peptide (Agro-Bio) used to produce the antibody (from 0 to 2.5 μg/mL). The blots were stripped and re-probed with antibodies against vinculin (VCL) used as the loading control. (TIF 93 kb)

Additional file 2: **Table S1.** Characteristics of primary antibodies used for western blotting and / or immunohistochemistry or immunofluorescence. (DOCX 16 kb)

Additional file 3: **Figure S2.** Experiment design of the study. * Some experiments enabled to measure both progesterone and estradiol in supernatants of the same 96-well dishes. (TIF 342 kb)

Additional file 4: **Figure S3.** Gene expression of (A) free fatty acid receptor 1 (*FFAR1*) and (B) free fatty acid receptor 4 (*FFAR4*) in bovine ovarian cells. Total mRNA was extracted from the ovarian cortex (CX), thecal cells (TH), granulosa cells (GC) and cumulus cells (CC). Total mRNA was then reverse-transcribed and real-time RT-PCR was performed. The geometric mean of two housekeeping genes (*RPL19*- ribosomal protein L19 and *RPS9*- ribosomal protein S9) was used to normalize gene expression. Results of 2 to 4 independent samples are presented as means ± SEM. Bars with different superscripts are significantly different (*p* < 0.05). (TIF 59 kb)

Additional file 5: **Figure S4.** Expression and localization of free fatty acid receptor 4 (FFAR4) in bovine granulosa cells by immunofluorescence with a commercial antibody against human FFAR4. Immunofluorescence was performed on granulosa cells (GC) after in vitro culture. Briefly, recovered GCs after follicle puncture and GC washing were incubated in serum-free modified McCoy’s 5A medium (2.5 × 10^5^ viable cells/well on a 8-well chamber slide (Lab-Tek® Nunc) for 48 h. Cultures were performed in a water-saturated atmosphere containing 5% CO_2_ in air at 38 °C. FFAR4 (green fluorescence) was immunodetected in GC (commercial human FFAR4 rabbit antibody, Aviva Systems Biology, Clinisciences, Nanterre, France) with a similar protocol to the protocol used with the customized anti-FFAR4 antibody. The commercial anti- FFAR4 antibody was produced by using a peptide from the FFAR4 human c-terminal region, which shares 89% identity (Protein BLAST® result on NCBI website) with the *Bos taurus* FFAR4 (Accession number: NP_001315586.1) and no identity with other amino acid sequences of bovine proteome. Rabbit IgG was used as the control with the same secondary antibody as for FFAR4 detection. Nuclei were stained with Hoechst 33,258 (blue fluorescence). Fluorescence was observed under a Zeiss confocal microscope LSM700 (Carl Zeiss Microscopy GmbH, Munich, Germany) using an oil 63× objective and the appropriate filters. The images were captured using Zen 2012 software (black edition version 8.0, Carl Zeiss Microscopy GmbH). The picture framed in red is a magnification of the area framed in red from the original image. Bars = 10 μm. (TIF 132 kb)

Additional file 6: **Figure S6.** Protein expression of (A) proliferating cell nuclear antigen (PCNA), (B) hydroxy-delta-5-steroid dehydrogenase, 3 beta- and steroid delta-isomerase 1 (HSD3B1), (C) steroidogenic acute regulatory protein (StAR) and (D) cytochrome P450 family 11 subfamily A member 1 (CYP11A1) after 15 h treatment with DHA. Effects of DHA treatment on protein levels were assessed in bovine granulosa cells after 15 h culture in enriched McCoy’s 5A media in presence or absence of DHA 10 or 20 μM. The chemical DMSO alone (1/2000) was used as a negative control due to its solvent activity on DHA. Protein extracts were separated by electrophoresis on 4–12% (w:v) SDS-polyacrylamide gel. After electrotransfer to nitrocellulose membranes, the proteins were probed with anti-PCNA (A), anti-HSD3B1 (B), anti-StAR (C) or anti-CYP11A1 (D) antibodies. The blots were stripped and re-probed with antibodies against Vinculin (VCL). The blots presented are representative of the quantification reported in Fig. 6. (TIF 180 kb)

Additional file 7: **Figure S5.** Signaling pathways in bovine granulosa cells after treatment with other concentrations of DHA (50 μM) or TUG-891 (10 and 50 μM). Effects of DHA or TUG-891 on phosphorylation of (A) mitogen-activated protein kinase 14 (MAPK14), (B) AMP-activated protein kinaseα (AMPKα) and (C) mitogen-activated protein kinase 1/3 (MAPK1/3) signaling pathways were assessed in bovine granulosa cells cultured for 15 h in enriched McCoy’s 5A media with 50 μM DHA or with 10 or 50 μM TUG-891, as described in Material and Method section for 5, 10, 30 and 60 min. Protein extracts were separated by electrophoresis on 4–12% (w:v) SDS-polyacrylamide gel. After electrotransfer to nitrocellulose membranes, the proteins were probed with anti-phosphorylated (p-)MAPK14 (A), anti-p-AMPKα (B) or anti-p-MAPK1/3 (C) antibodies. The blots were stripped and re-probed with antibodies against MAPK14, AMPKα, or MAPK1/3, respectively. Bands on the blots were quantified. Results of four independent experiments are presented as the ratio of p-protein to total protein, normalized by the ratio observed in control at each time and expressed as mean ± SEM, with time 0 min being equal to 1 (for reference). Bars with different superscripts are significantly different (*p* < 0.05). (TIF 99 kb)

Additional file 8: **Figure S7.** Signaling pathways in bovine granulosa cells after DHA or TUG-891 treatment. Effects of DHA or TUG-891 on phosphorylation of (A) mitogen-activated protein kinase 14 (MAPK14), (B) AMP-activated protein kinaseα (AMPKα) and (C) protein kinase B (Akt) signaling pathways were assessed in bovine granulosa cells cultured for 15 h in enriched McCoy’s 5A media with 10 μM DHA or 1 μM TUG-891, as described in Material and Method section for 5, 10, 30 and 60 min. Protein extracts were separated by electrophoresis on 4–12% (w:v) SDS-polyacrylamide gel. After electrotransfer to nitrocellulose membranes, the proteins were probed with anti-phosphorylated (p-) MAPK14 (A), anti-p-AMPKα (B) or anti-p-AKT1/2/3 (C) antibodies. The blots were stripped and re-probed with antibodies against MAPK14, AMPKα or Akt, respectively. The blots presented are representative of the quantification reported in Fig. 8. (TIF 228 kb)

## Abbreviations

Akt: Protein kinase B
ALA: Alpha-linolenic acid
AMPKα: AMP-activated protein kinaseα
BSA: Bovine serum albumin
CYP11A1: Cytochrome P450 family 11 subfamily A member 1
DAB: Diaminobenzidine tetrahydrochloride dehydrate
DHA: Docosahexaenoic acid
DMSO: Dimethyl sulfoxide
DNA: Deoxyribonucleic acid
ELISA: Enzyme-linked immunosorbent assay
EPA: Eicosapentaenoic acid
FA: Fatty acids
FAME: Fatty acid methyl ester
FFAR1: Free fatty acid membrane receptor 1
FFAR4: Free fatty acid membrane receptor 4
GC: Granulosa cells
GLUT1: Glucose Transporter Type 1
GPX4: Glutathione peroxidase 4
HRP: Horseradish peroxidase
HSD3B1: Hydroxy-delta-5-steroid dehydrogenase, 3 beta- and steroid delta-isomerase 1
IgG: Immunoglobulin G
IL-1β: Interleukine 1 beta
IL-6: Interleukine 6
IL-8: Interleukine 8
IκB: I kappa B
LDL: Low density lipoprotein
MAP K14: Mitogen-Activated Protein Kinase 14
MAPK1/3: Mitogen-Activated Protein Kinase 1/3
MCP-1: Monocyte Chemoattractant Protein-1
NFDMP: Non-fat dry milk powder
NFkB: Nuclear factor kappa B
PBS: Phosphate buffered saline
PCNA: Proliferating cell nuclear antigen
PCR: Polymerase chain reaction
PGF2α: Prostaglandin F2 alpha
PPARs: Peroxisome proliferator-activated receptors
PTGS2: Prostaglandin-Endoperoxide Synthase 2
PUFA: Polyunsaturated fatty acid
RNA: Ribonucleic acid
RPL19: Ribosomal protein L19
RPS9: Ribosomal protein S9
SEM: Standard error of the mean
SREBF1: Sterol Regulatory Element Binding Transcription Factor 1
StAR: Steroidogenic acute regulatory protein
TBS: Tris-buffer saline
TGFβ1: Transforming growth factor beta1
TNF: Tumor necrosis factor
