# Supplementary figures and images for: Docosahexaenoic acid (DHA) effects on proliferation and steroidogenesis of bovine granulosa cells
Source: Reprod Biol Endocrinol. 2018 Apr 26;16:40. doi: 10.1186/s12958-018-0357-7 (PMC5918968; doi:10.1186/s12958-018-0357-7)

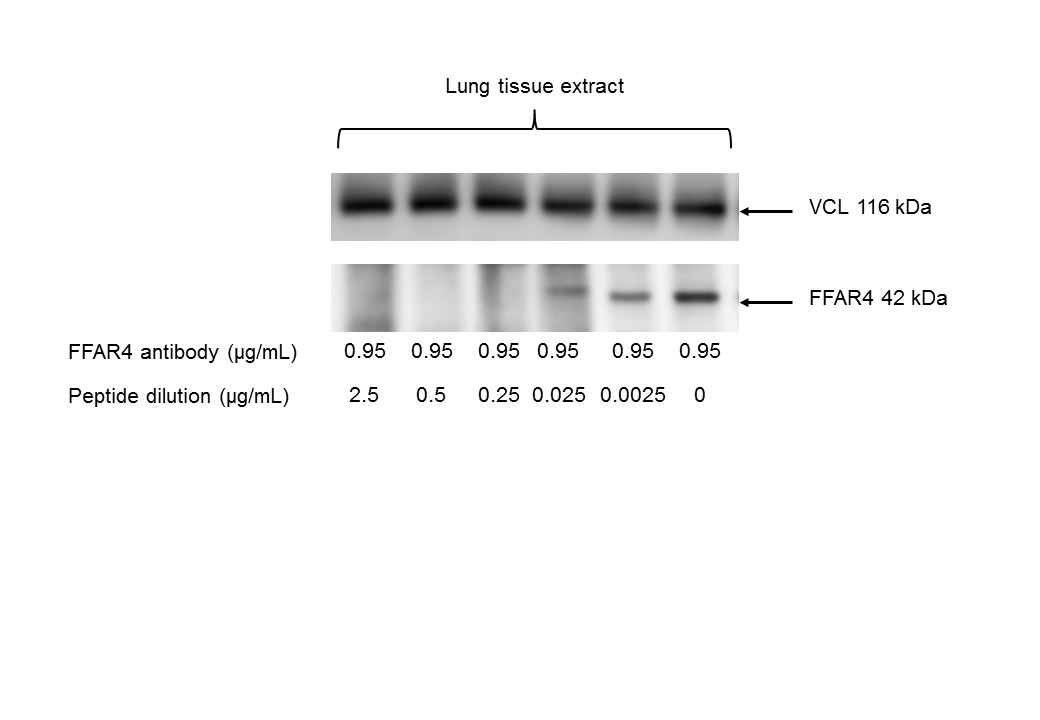

Supplement: Supplementary file 1 — Figure S1. Control of customized free fatty acid receptor 4 (FFAR4) antibody specificity. Protein extracts from bovine lung tissue were separated by electrophoresis on 4–12% (w:v) SDS-polyacrylamide gel. After electrotransfer to nitrocellulose membranes, the proteins were probed with anti-FFAR4 antibody (0.95 μg/mL, customized FFAR4 rabbit antibody, Agro-Bio), which was pre-incubated for 15 min with different concentrations of the bovine specific peptide (Agro-Bio) used to produce the antibody (from 0 to 2.5 μg/mL). The blots were stripped and re-probed with antibodies against vinculin (VCL) used as the loading control. (TIF 93 kb) [file 12958_2018_357_MOESM1_ESM.tif]

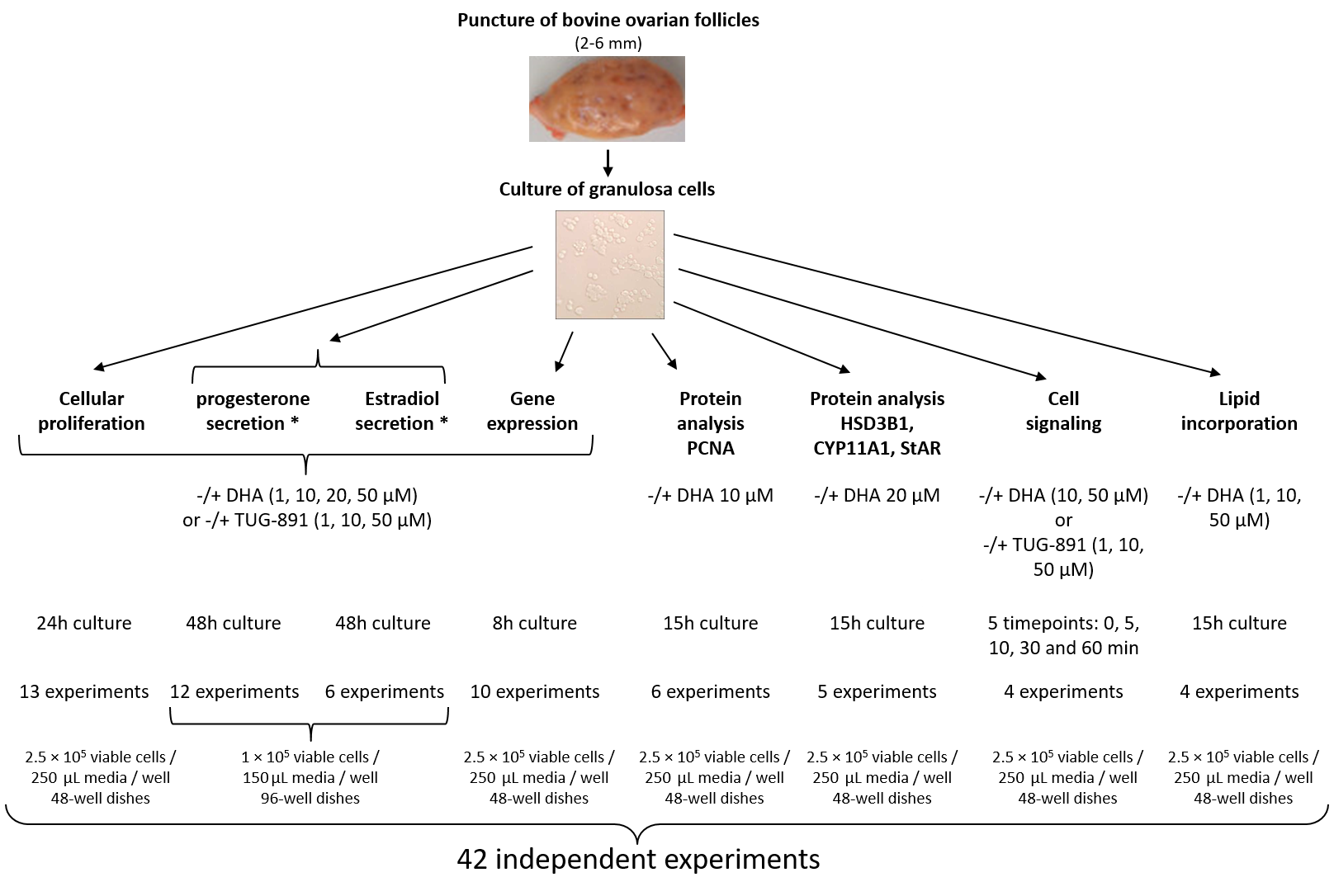

Supplement: Supplementary file 3 — Figure S2. Experiment design of the study. * Some experiments enabled to measure both progesterone and estradiol in supernatants of the same 96-well dishes. (TIF 342 kb) [file 12958_2018_357_MOESM3_ESM.tif]

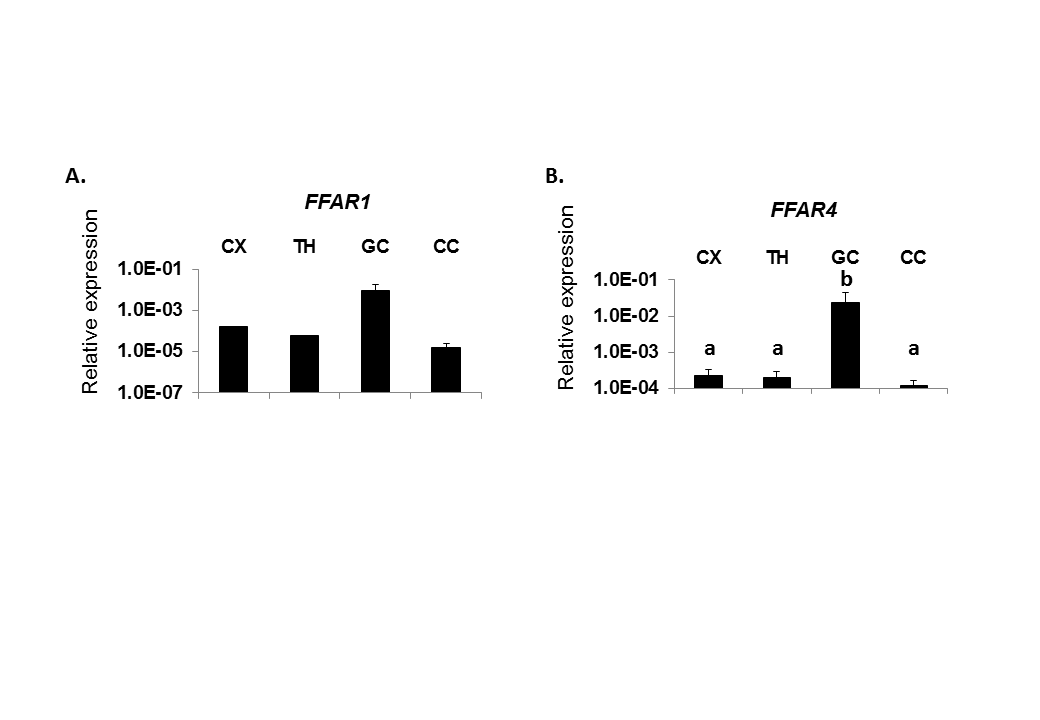

Supplement: Supplementary file 4 — Figure S3. Gene expression of (A) free fatty acid receptor 1 (FFAR1) and (B) free fatty acid receptor 4 (FFAR4) in bovine ovarian cells. Total mRNA was extracted from the ovarian cortex (CX), thecal cells (TH), granulosa cells (GC) and cumulus cells (CC). Total mRNA was then reverse-transcribed and real-time RT-PCR was performed. The geometric mean of two housekeeping genes (RPL19- ribosomal protein L19 and RPS9- ribosomal protein S9) was used to normalize gene expression. Results of 2 to 4 independent samples are presented as means ± SEM. Bars with different superscripts are significantly different (p < 0.05). (TIF 59 kb) [file 12958_2018_357_MOESM4_ESM.tif]

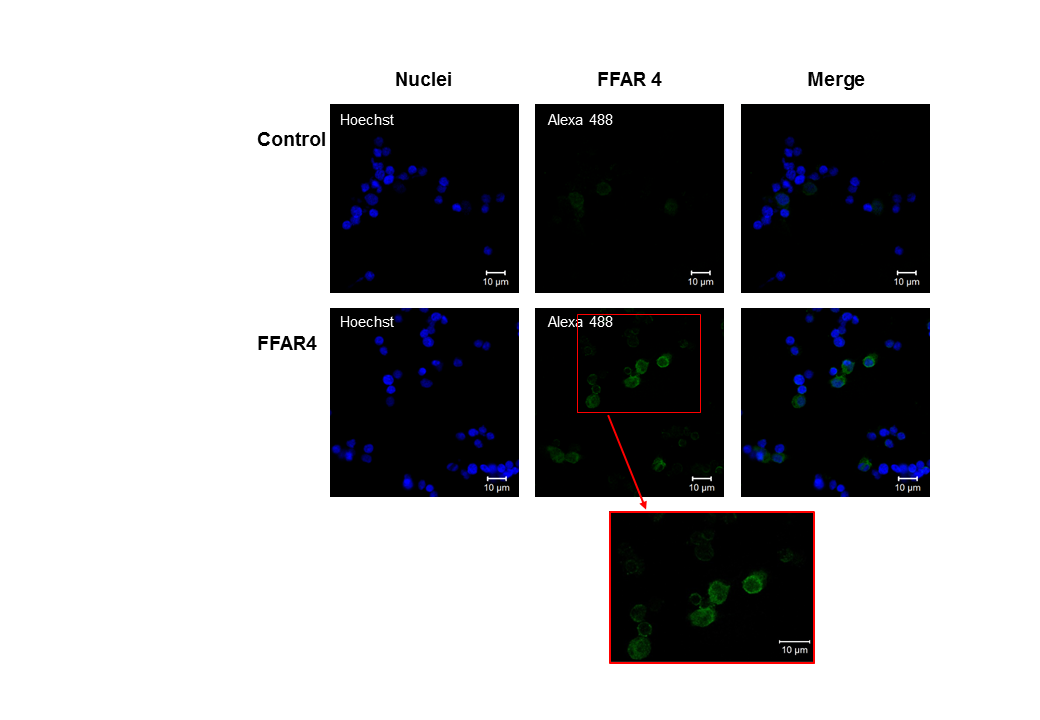

Supplement: Supplementary file 5 — Figure S4. Expression and localization of free fatty acid receptor 4 (FFAR4) in bovine granulosa cells by immunofluorescence with a commercial antibody against human FFAR4. Immunofluorescence was performed on granulosa cells (GC) after in vitro culture. Briefly, recovered GCs after follicle puncture and GC washing were incubated in serum-free modified McCoy’s 5A medium (2.5 × 10^5^ viable cells/well on a 8-well chamber slide (Lab-Tek® Nunc) for 48 h. Cultures were performed in a water-saturated atmosphere containing 5% CO2 in air at 38 °C. FFAR4 (green fluorescence) was immunodetected in GC (commercial human FFAR4 rabbit antibody, Aviva Systems Biology, Clinisciences, Nanterre, France) with a similar protocol to the protocol used with the customized anti-FFAR4 antibody. The commercial anti- FFAR4 antibody was produced by using a peptide from the FFAR4 human c-terminal region, which shares 89% identity (Protein BLAST® result on NCBI website) with the Bos taurus FFAR4 (Accession number: NP_001315586.1) and no identity with other amino acid sequences of bovine proteome. Rabbit IgG was used as the control with the same secondary antibody as for FFAR4 detection. Nuclei were stained with Hoechst 33,258 (blue fluorescence). Fluorescence was observed under a Zeiss confocal microscope LSM700 (Carl Zeiss Microscopy GmbH, Munich, Germany) using an oil 63× objective and the appropriate filters. The images were captured using Zen 2012 software (black edition version 8.0, Carl Zeiss Microscopy GmbH). The picture framed in red is a magnification of the area framed in red from the original image. Bars = 10 μm. (TIF 132 kb) [file 12958_2018_357_MOESM5_ESM.tif]

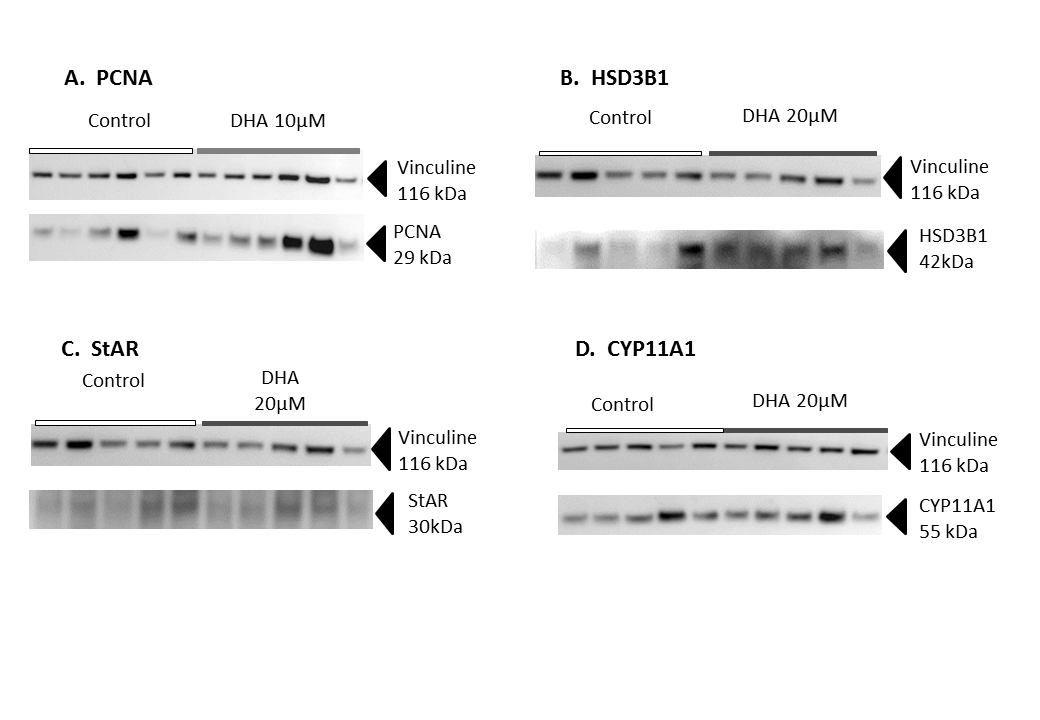

Supplement: Supplementary file 6 — Figure S6. Protein expression of (A) proliferating cell nuclear antigen (PCNA), (B) hydroxy-delta-5-steroid dehydrogenase, 3 beta- and steroid delta-isomerase 1 (HSD3B1), (C) steroidogenic acute regulatory protein (StAR) and (D) cytochrome P450 family 11 subfamily A member 1 (CYP11A1) after 15 h treatment with DHA. Effects of DHA treatment on protein levels were assessed in bovine granulosa cells after 15 h culture in enriched McCoy’s 5A media in presence or absence of DHA 10 or 20 μM. The chemical DMSO alone (1/2000) was used as a negative control due to its solvent activity on DHA. Protein extracts were separated by electrophoresis on 4–12% (w:v) SDS-polyacrylamide gel. After electrotransfer to nitrocellulose membranes, the proteins were probed with anti-PCNA (A), anti-HSD3B1 (B), anti-StAR (C) or anti-CYP11A1 (D) antibodies. The blots were stripped and re-probed with antibodies against Vinculin (VCL). The blots presented are representative of the quantification reported in Fig. 6. (TIF 180 kb) [file 12958_2018_357_MOESM6_ESM.tif]

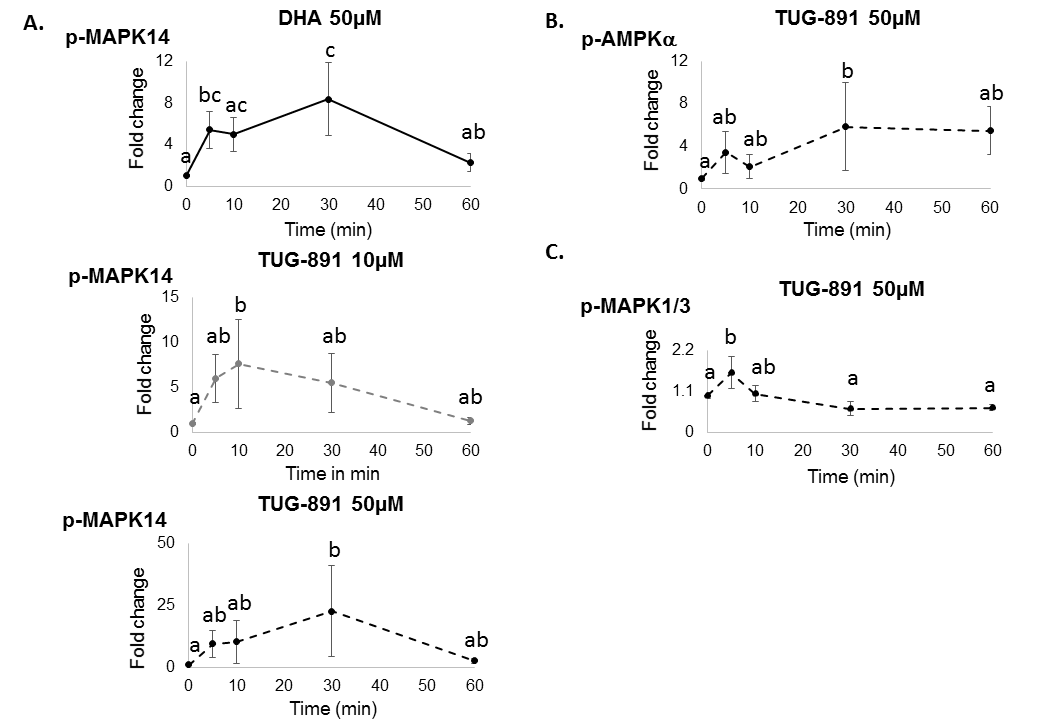

Supplement: Supplementary file 7 — Figure S5. Signaling pathways in bovine granulosa cells after treatment with other concentrations of DHA (50 μM) or TUG-891 (10 and 50 μM). Effects of DHA or TUG-891 on phosphorylation of (A) mitogen-activated protein kinase 14 (MAPK14), (B) AMP-activated protein kinaseα (AMPKα) and (C) mitogen-activated protein kinase 1/3 (MAPK1/3) signaling pathways were assessed in bovine granulosa cells cultured for 15 h in enriched McCoy’s 5A media with 50 μM DHA or with 10 or 50 μM TUG-891, as described in Material and Method section for 5, 10, 30 and 60 min. Protein extracts were separated by electrophoresis on 4–12% (w:v) SDS-polyacrylamide gel. After electrotransfer to nitrocellulose membranes, the proteins were probed with anti-phosphorylated (p-)MAPK14 (A), anti-p-AMPKα (B) or anti-p-MAPK1/3 (C) antibodies. The blots were stripped and re-probed with antibodies against MAPK14, AMPKα, or MAPK1/3, respectively. Bands on the blots were quantified. Results of four independent experiments are presented as the ratio of p-protein to total protein, normalized by the ratio observed in control at each time and expressed as mean ± SEM, with time 0 min being equal to 1 (for reference). Bars with different superscripts are significantly different (p < 0.05). (TIF 99 kb) [file 12958_2018_357_MOESM7_ESM.tif]

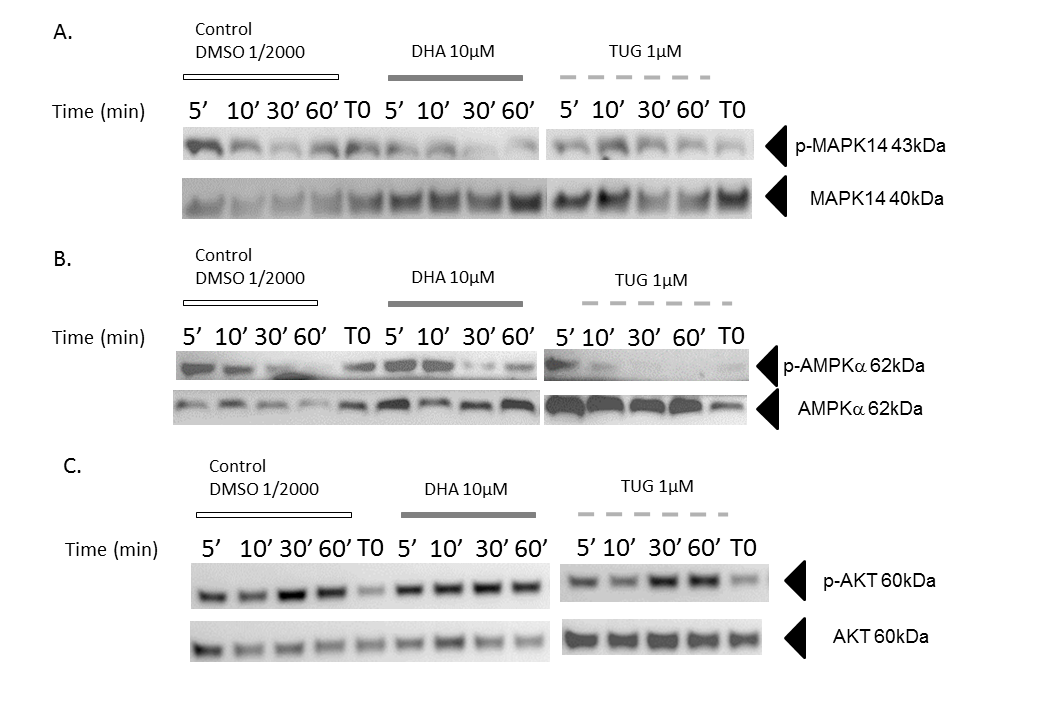

Supplement: Supplementary file 8 — Figure S7. Signaling pathways in bovine granulosa cells after DHA or TUG-891 treatment. Effects of DHA or TUG-891 on phosphorylation of (A) mitogen-activated protein kinase 14 (MAPK14), (B) AMP-activated protein kinaseα (AMPKα) and (C) protein kinase B (Akt) signaling pathways were assessed in bovine granulosa cells cultured for 15 h in enriched McCoy’s 5A media with 10 μM DHA or 1 μM TUG-891, as described in Material and Method section for 5, 10, 30 and 60 min. Protein extracts were separated by electrophoresis on 4–12% (w:v) SDS-polyacrylamide gel. After electrotransfer to nitrocellulose membranes, the proteins were probed with anti-phosphorylated (p-) MAPK14 (A), anti-p-AMPKα (B) or anti-p-AKT1/2/3 (C) antibodies. The blots were stripped and re-probed with antibodies against MAPK14, AMPKα or Akt, respectively. The blots presented are representative of the quantification reported in Fig. 8. (TIF 228 kb) [file 12958_2018_357_MOESM8_ESM.tif]
