# Supplementary material for: Docosahexaenoic acid (DHA) effects on proliferation and steroidogenesis of bovine granulosa cells
Source: Reprod Biol Endocrinol. 2018 Apr 26;16:40. doi: 10.1186/s12958-018-0357-7 (PMC5918968; doi:10.1186/s12958-018-0357-7)
Supplement: Supplementary file 2 — Table S1. Characteristics of primary antibodies used for western blotting and / or immunohistochemistry or immunofluorescence. (DOCX 16 kb) [file 12958_2018_357_MOESM2_ESM.docx]

Additional file 2: Table S1 Characteristics of primary antibodies used for western blotting and / or immunohistochemistry or immunofluorescence

| **Antibody name** | **Dilution ratio** | **Specie specificity** | **Source** | **Supplier (distributor, town, country)** | **Reference** |
| --- | --- | --- | --- | --- | --- |
| **PKB** (AKT) | 1:1000 | Mouse | Rabbit pAb | Cell Signaling Technology (Ozyme, Saint Quentin Yvelines, France) | 9272 |
| **p-AKT** (Ser473, D9E, XP^TM^) | 1:2000 | Human | Rabbit mAb | Cell Signaling Technology | 4060 |
| **AMPKα** | 1:1000 | Human | Rabbit pAb | Cell Signaling Technology | 2532 |
| **p-AMPKα** (Thr172) | 1:1000 | Human | Rabbit pAb | Cell Signaling Technology | 2531 |
| **FFAR4** | 1:50-1:1000 | Bovine | Rabbit pAb | Agro-Bio (La Ferté Saint-Aubin, France) | Customized Ab |
| **FFAR4** (O3FAR1) | 1:50 | Human | Rabbit pAb | Aviva Systems Biology (Clinisciences, Nanterre, France) | ARP62558_P050 |
| **p44/42 MAPK** (MAPK1/3, ERK1/2) | 1:1000 | Rat | Rabbit pAb | Cell Signaling Technology | 9102 |
| **p-p44/42 MAPK** (MAPK1/3, ERK1/2) (Thr^202^/Tyr^204^, D13.14.4E, XP^TM^) | 1:2000 | Human | Rabbit mAb | Cell Signaling Technology | 4370 |
| **p38 MAPK** (MAPK14, D13E1, XP^TM^) | 1:1000 | Human | Rabbit mAb | Cell Signaling Technology | 8690 |
| **p-p38 MAPK** (MAPK14, Thr^180^/Tyr^182^, D3F9, XP^TM^) | 1:1000 | Human | Rabbit mAb | Cell Signaling Technology | 4511 |
| **PCNA** | 1:500 | Rat | Mouse mAb | Millipore (Temecula, California, United States) | MAB424 |
| **HSD3B1** (3β-HSD) | 1:500 | Human | Rabbit pAb | Abgent (San Diego, California, United States) | AP14585a |
| **CYP11A1** (CytP450scc) | 1:500 | Human | Goat pAb | Santa Cruz Biotechnology (Euromedex, France) | Sc-18043 |
| **StAR** (FL-285) | 1:500 | Human | Rabbit pAb | Santa Cruz Biotechnology | Sc-25806 |
| **Vinculin** | 1:1000 | Human | Mouse mAb | Sigma-Aldrich (Saint Quentin Fallavier, France) | V9131 |

PKB, protein kinase B; p-, phosphorylated; AMPKα, AMP-activated protein kinaseα; FFAR4, free fatty acid receptor 4; MAPK, mitogen-activated protein kinase; ERK, extracellular signal-regulated kinase; PCNA, proliferating cell nuclear antigen; HSD3B1, hydroxy-delta-5-steroid dehydrogenase, 3 beta- and steroid delta-isomerase 1; CYP11A1, cytochrome P450 family 11 subfamily A member 1; StAR, steroidogenic acute regulatory protein; pAb, polyclonal antibody; mAb, monoclonal antibody.
